# Simultaneous inhibition of cholinesterase and antagonism of histamine H3 receptors alleviates cognitive deficits and mitigates apoptosis in scopolamine-induced amnesia in mice

**DOI:** 10.3389/fnbeh.2026.1722019

**Published:** 2026-01-29

**Authors:** Wajd Ebdah, Shilu Deepa Thomas, Nermin Eissa, Petrilla Jayaprakash, Dorota Łażewska, Katarzyna Kieć-Kononowicz, Bassem Sadek

**Affiliations:** 1 Department of Pharmacology and Therapeutics, College of Medicine and Health Sciences, United Arab Emirates University, Al Ain, United Arab Emirates; 2 Zayed Center for Health Sciences, United Arab Emirates University, Al Ain, United Arab Emirates; 3 Department of Biomedical Sciences, College of Health Sciences, Abu Dhabi University, Abu Dhabi, United Arab Emirates; 4 Department of Technology and Biotechnology of Drugs, Faculty of Pharmacy, Jagiellonian University-Medical College, Kraków, Poland

**Keywords:** apoptosis, ChEI, E100, H3R antagonist, neuroinflammation, oxidative stress

## Abstract

**Introduction:**

Alzheimer’s disease (AD) is a progressive neurodegenerative disorder characterized by cognitive decline and memory deficits. Mounting evidence highlights the role of cholinergic and histaminergic neurotransmissions in the pathophysiology of AD. Hence, developing agents that target multiple neurotransmitter systems may provide improved therapeutic benefits.

**Methods:**

This study investigated the effects of acute systemic administration of E100, a dual-active cholinesterase inhibitor (ChEI) and histamine H3 receptor (H3R) antagonist, on scopolamine (SCO)-induced memory impairment in male C57BL/6 mice. Behavioral assessments, including the Novel Object Recognition Test (NORT), Y-Maze test (YMT), Three-Chamber Test (TCT), Fear Conditioning test (FCT), and Elevated Plus Maze (EPM), were conducted to assess cognitive performance while biochemical analyses assessed apoptotic markers, oxidative stress, neuroinflammation and acetylcholinesterase activity.

**Results:**

Systemic administration of E100 (10 mg/kg, i.p.) significantly improved memory function in SCO-induced amnesia, as evidenced by enhanced short-term memory (STM) (*p* < 0.001) and long-term memory (LTM) (*p* < 0.01) performance in the NORT, as well as improved spatial memory in YMT (*p* < 0.001) and FCT (*p* < 0.001; for cued fear memory) and (*p* < 0.001; for contextual fear memory). Additionally, E100 treatment in the TCT, improved social memory (*p* < 0.001) and alleviated SCO-induced anxiety-related deficits in the EPM (*p* < 0.001). Moreover, treatment with E100 (10 mg/kg, i.p.) attenuated SCO-induced neuroinflammation by reducing TNF-α and IL-1β levels and mitigated oxidative stress by increasing GSH and SOD while decreasing MDA levels in the hippocampus and cerebellum (*p*’s < 0.001). E100 also reduced caspase-1 activity (*p* < 0.001), suggesting its anti-apoptotic effect. Furthermore, E100 attenuated the elevated AChE activity observed in SCO-induced amnesic mice (*p* < 0.01), providing effects comparable to those of the reference drug Donepezil.

**Discussion:**

These findings provide extensive *in vivo* evidence of the neuroprotective effects of E100, demonstrating its ability to ameliorate memory deficits, mitigate neuroinflammation and restore oxidative as well as AChE activity balance. By targeting both cholinergic and histaminergic dysfunction in the brain, E100 offers a promising therapeutic strategy for AD and related neurodegenerative disorders. This study highlights the potential role of dual-active ChEIs and H3R antagonists in memory impairment, and addressing multiple neuropathological mechanisms underlying AD.

## 1 Introduction

Alzheimer’s disease (AD) is a progressive neurodegenerative disorder characterized by the accumulation of extracellular amyloid-β (Aβ) plaques and intracellular neurofibrillary tangles composed of hyperphosphorylated tau protein. These pathological hallmarks are accompanied by neuroinflammation, synaptic damage, neuronal death and cortical atrophy, ultimately leading to cognitive decline (Long and Holtzman, 2019; Wang et al., 2022). Activated microglia and astrocytes play a central role in AD by releasing proinflammatory cytokines such as IL-1β and TNF-α, which exacerbate Aβ accumulation and neuronal damage (Garland et al., 2022; Merighi et al., 2022). Clinically, AD is the leading cause of dementia and is marked by progressive impairments in episodic memory, decision-making and spatial orientation (Blennow et al., 2006). The cholinergic hypothesis is one of the earliest theories to explain the development of AD, with cholinergic neurotransmission playing a central role (Hampel et al., 2019). Acetycholine (ACh) is essential for memory, learning, attention, and stress responses. Numerous studies have demonstrated that individuals with age-related memory decline and AD exhibit a marked loss of cholinergic neurons in the hippocampus, accompanied by a significant reduction in ACh levels (Huang et al., 2022). In AD, reduced cholinergic neurotransmission, especially due to decreased activity of choline acetyltransferase, an enzyme involved in ACh synthesis, in brain regions like the hippocampus and cortex, contributes to memory deficits (Chen and Mobley, 2019). ACh also regulates immune responses via the cholinergic anti-inflammatory pathway, helping to suppress neuroinflammation, a key feature of AD (Han et al., 2017). Cholinesterase (ChE) breaks down ACh released at synapses in the CNS. Acetylcholinesterase (AChE) directly interact with presenilin-1, a key enzyme involved in the production of Aβ, thus promoting Aβ accumulation and cognitive decline (Campanari et al., 2014; Chen et al., 2022). Acetylcholinesterase inhibitors (AChEIs) such as donepezil have been developed and are widely used to manage AD symptoms by enhancing cholinergic transmission (Martins et al., 2018). Moreover, postmortem and experimental studies have shown a significant decrease in histamine (HA) across different brain regions, linked to reduced expression of histaminergic receptors (Zlomuzica et al., 2016). Brain HA exerts its effects through four G protein-coupled receptors (GPCRs), namely H1R, H2R, H3R, and H4R. The H3Rs are primarily found in the presynaptic neurons, where they act as auto-receptors modulating the synthesis and release of HA. Additionally, H3Rs also act as hetero-receptors, influencing additional neurotransmitters such as GABA, dopamine, glutamate, serotonin, norepinephrine, and ACh (Medhurst et al., 2009; Panula and Nuutinen, 2013). H3Rs also influence neuroinflammatory pathways, making them potential therapeutic targets for neurodegenerative diseases, e.g., AD. Therefore, blockage of H3Rs can reduce microglial activation, mitigate neuroinflammation, and exert neuroprotective effects by reducing oxidative stress and enhancing HA release (Carthy and Ellender, 2021; Eissa et al., 2022a; Thomas et al., 2024b). Scopolamine (SCO) is commonly used to induce amnesia in experimental animals by blocking muscarinic receptors, which results in an increase in AChE activity in cortical and hippocampal neurons, causing impairments in memory and learning mimicking AD like pathology (Chen et al., 2014; Xu et al., 2016; Song et al., 2017; Al Omairi et al., 2019). SCO reduces hippocampal neurogenesis and cholinergic activity, while promoting oxidative stress through increased lipid peroxidation and ROS production (Tang, 2019; Mishra and Thakur, 2023). Furthermore, SCO induces apoptosis via mitochondrial dysfunction and activation of pro-apoptotic markers like Bax and caspase-3 (Ajami et al., 2012; Jahanshahi et al., 2013; Kim et al., 2018). SCO model is useful for studying AD-related cognitive decline and testing potential therapeutic interventions (Haider et al., 2016).

**FIGURE 1:**
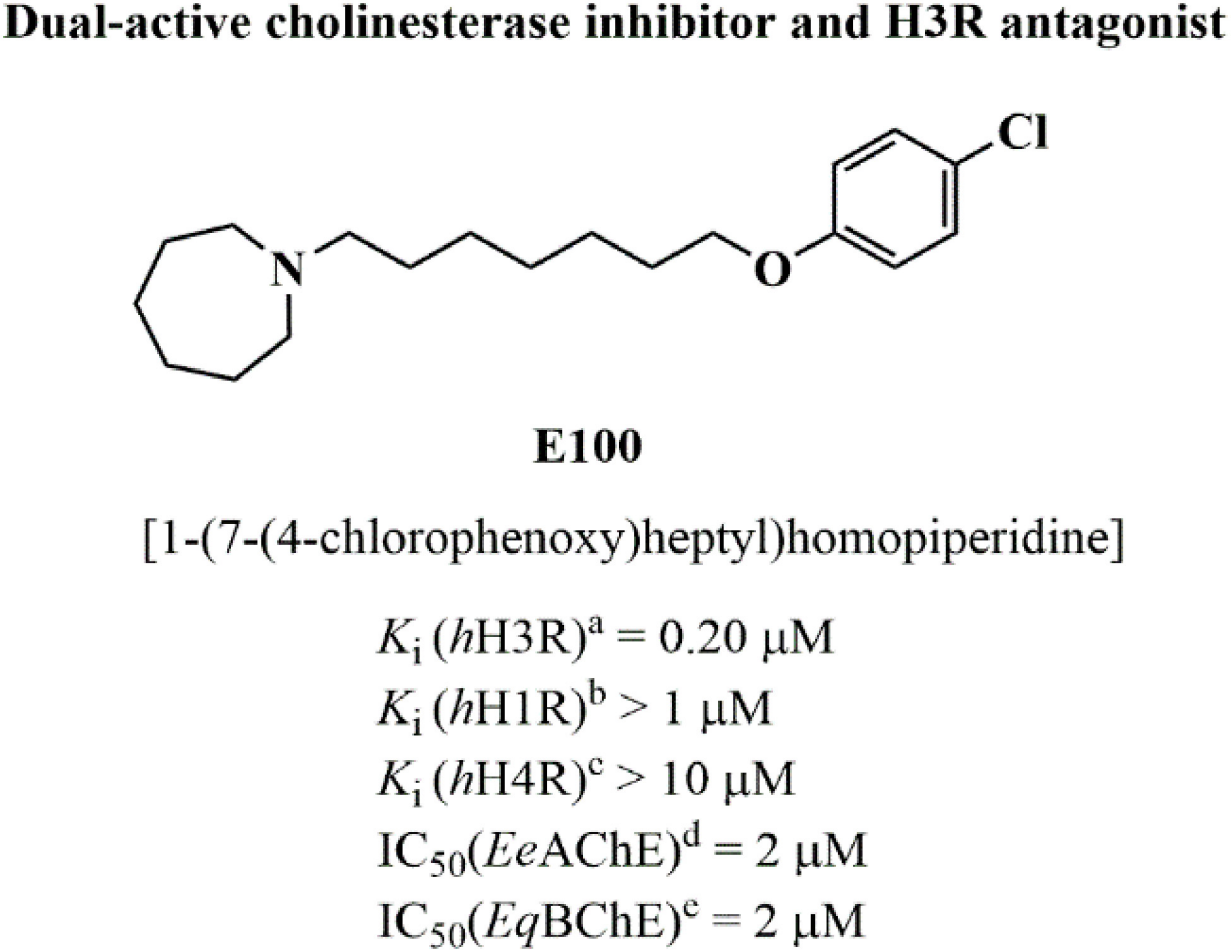
Chemical structure and *in vitro* affinities of E100 on human histamine receptor subtypes and choline esterase enzymes. ^a–c^Binding assays were conducted to evaluate the affinity of the compounds to H1-, H3-, and H4 receptors. Assays were performed using cells expressing the respective receptors, following previously established protocols (Kuder et al., 2016; Łażewska et al., 2016; Eissa et al., 2019, Eissa et al., 2020a). ^d^AChE, Acetylcholineesterase; *Ee*, electric eel. ^e^BuChE, Butyrylcholinesterase; *Eq*, equine.

Currently, FDA-approved drugs available for AD include the AChEIs (donepezil, rivastigmine, and galantamine) as well as the NMDA receptor antagonist memantine. These drugs provide only symptomatic relief by partially stabilizing cognitive functions and managing behavioral and psychological symptoms of dementia. However, their efficacy is modest and limited to slowing symptom progression rather than modifying the underlying disease pathology (Passeri et al., 2022). Consequently, these treatments improve quality of life but do not halt or reverse disease progression. Pitolisant is the main H3 receptor antagonist/inverse agonist currently used clinically for treating excessive daytime sleepiness in adults with narcolepsy. Several H3 antagonists/inverse agonists have been tested for conditions such as Alzheimer’s and Parkinson’s disease; however, none have been approved for clinical use (Thomas et al., 2024a). Due to the AD’s complexity, targeting a single receptor is insufficient. Hence, multitarget-directed ligands (MTDLs) are being developed to simultaneously target multiple pathways, particularly for treating neurodegenerative disorders, with growing interest in compounds that block H3Rs as well enhance ACh levels (Sharma, 2019; Lopes et al., 2021). Currently, there are no clinically approved drugs that simultaneously target both cholinesterase inhibition and H3 receptor antagonism, underscoring the novelty and therapeutic potential of dual-acting compounds like E100. Thus, in the current study, the mitigating effects of a recently developed dual-active cholinesterase inhibitor and H3R antagonist, namely E100 on associative, social, and recognition memory and on brain caspase-1 activity, neuroinflammation, acetylcholine esterase activity, and oxidative stress in male C57BL/6 mice model of SCO-induced amnesia were evaluated, following acute systemic administration of a dose range (5, 10, and 15 mg/kg, i.p.) of E100 (Figure 1). E100 (1-(7-(4-chlorophenoxy)heptyl)azepine) exhibits balanced AChE inhibitory activity (*Ee*AChE: IC_50_ = 2 μM; *Eq*BChE: IC_50_ = 2 μM). It is further characterized as an H3R antagonist with an affinity of *h*H3R Ki = 0.2 μM and demonstrates a high selectivity profile for the H3R subtype. The effects of E100 on several types of memory and anxiety levels were also assessed through behavioral studies, while caspase-1 activity, acetylcholine esterase activity, neuroinflammation, and oxidative stress were evaluated in the hippocampus and cerebellum of treated mice using biochemical assessments. In addition, abrogation studies using (*R*)-α-methylhistamine (RAMH), a CNS-penetrant and selective H3R agonist, were conducted to further elucidate the role of histaminergic neurotransmission in the effects provided by E100 in improving cognitive function.

## 2 Materials and methods

### 2.1 Animals

Behavioral experiments were carried out using C57BL/6J (C57) mice. C57 male mice, aged 8–12 weeks and weighing between 25 and 28 grams, were obtained from the animal facility at the College of Medicine and Health Sciences, UAE University. All mice were kept in polypropylene cages (27 cm × 17 cm × 12 cm) with saw dust bedding in a controlled air-conditioned room (24 ± 2°C), with a 12-h light/dark cycle. Standard rodent chow and tap water were available *ad libitum*. Animal health was monitored daily by trained laboratory staff. Mice were randomly assigned to experimental groups. All experiments were carried out between 8 a.m. and 2 p.m. Male mice were chosen to reduce variability caused by hormonal fluctuations typically seen in female mice during their estrogen cycle. All experimental procedures were approved by the Institutional Animal Ethics Committee of the United Arab Emirates University (ERA-2017–5603) and conducted in compliance with EU guidelines (Directive 2010/63/EU) and ARRIVE (Animal Research: Reporting of *In Vivo* Experiments) guidelines for reporting animal research. Efforts were made to minimize both the number of animals used and any potential suffering. Furthermore, all behavioral assessments were conducted by the same experimenter.

### 2.2 Drugs and biochemical reagents

The test compound E100 was designed and synthesized in the Department of Technology and Biotechnology of Drugs, Krakow, Poland, according to previously described procedures (Kuder et al., 2016; Łażewska et al., 2016). Donepezil (DOZ, 1 mg/kg, i.p.), Pitolisant (PIT, 10 mg/kg i.p.), Scopolamine (SCO, 2 mg/kg i.p.), and the CNS-penetrant H3R agonist (*R*)-α-methylhistamine (RAMH, 10 mg/kg, i.p.) were purchased from Sigma-Aldrich (St. Louis, MO, United States). DOZ and PIT were used as standard reference drugs. All drugs were dissolved in isotonic saline solution and injected intraperitoneally (i.p.) at a volume of 10 ml/kg. The reduced glutathione (GSH) assay kit was sourced from Sigma-Aldrich (St. Louis, MO, United States). The lipid peroxidation assay kit for measuring malondialdehyde (MDA) was obtained from Northwest Life Science (Vancouver, WA, United States). Assay kits for superoxide dismutase (SOD) were acquired from Cayman Chemical (Ann Arbor, MI, United States). ELISA kits (DuoSet) for IL-1β and TNF-α were purchased from R&D Systems. The colorimetric assay kit for AChE activity (Product: ab65345) was purchased from BioVision, Milpitas, CA, USA.

### 2.3 Experimental design and procedure

**FIGURE 2:**
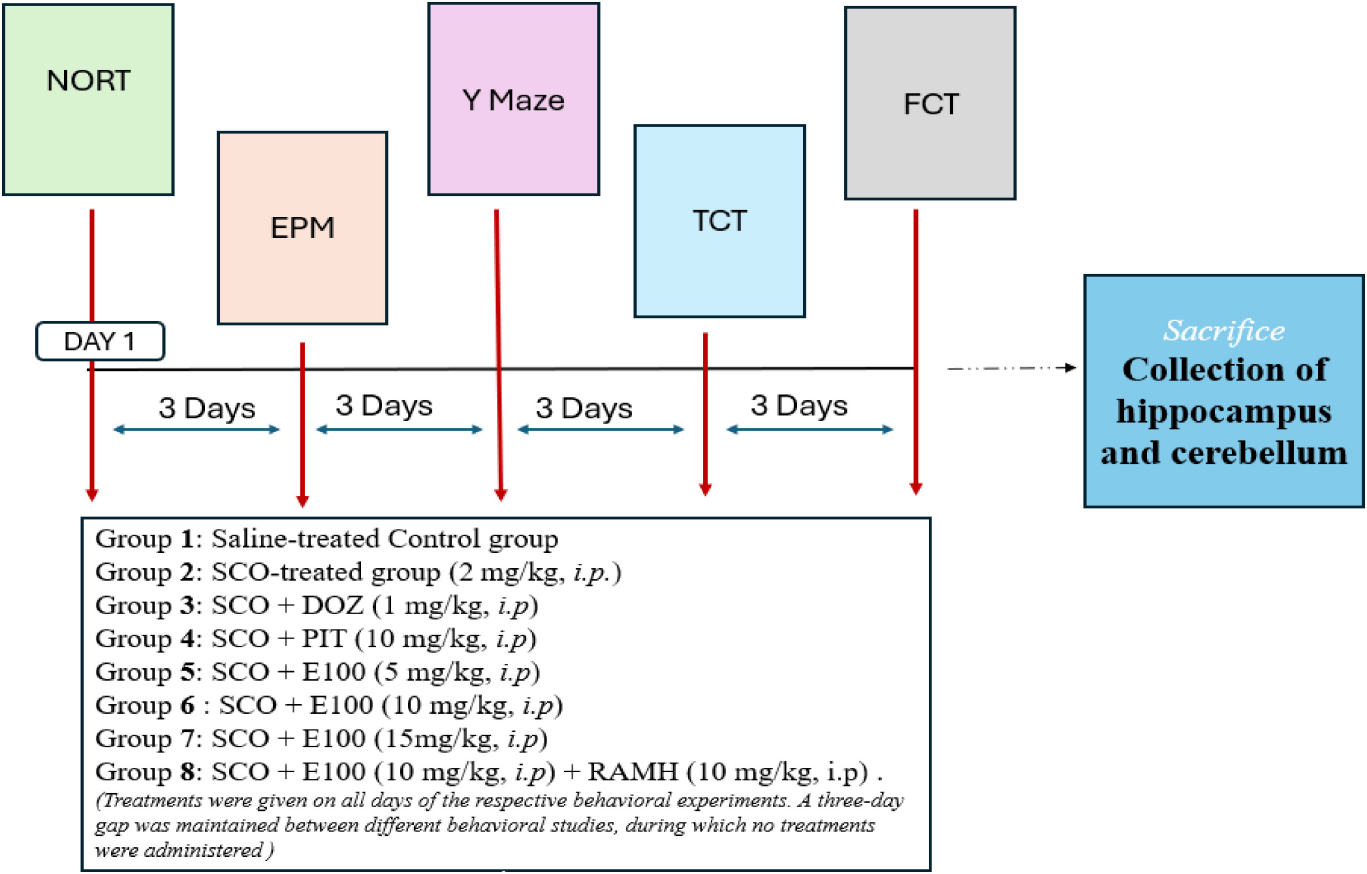
A graphical timeline for drug treatments and behavioral studies conducted in SCO-treated amnesic mice. SCO, Scopolamine, DOZ, Donepezil, PIT, Pitolisant, RAMH, (*R*)-α-methylhistamine.

The mice were assigned into eight groups: a vehicle-treated Control group, a SCO-treated group, a SCO + DOZ-treated positive control group, a SCO + PIT-treated positive control group, SCO + E100-treated groups, with 3 different dose (5, 10, and 15 mg/kg), and a SCO + E100 (best dose) + RAMH-treated group. Groups were comprised of six mice each. The dose range for dual active ChEI and H3R antagonist, E100 was determined based on previous studies where E100 was shown to improve autism-related behaviors and neuroinflammation in BTBR mice, as well as in mice with sodium valproate-induced autism (Eissa et al., 2019, 2020b). The doses for SCO, DOZ, PIT, and H3 receptor agonist RAMH were selected based on previously published experimental protocols (Kotańska et al., 2018; Cheon et al., 2021; Venkatachalam et al., 2021). Memory impairment was induced by i.p. injection of scopolamine (2 mg/kg) 30–45 min before each behavioral session. The control group received normal saline. E100 (5, 10, 15 mg/kg, i.p), DOZ (1 mg/kg, i.p) and PIT (10 mg/kg, i.p) were injected 15 min before each behavioral test. For the abrogation studies, E100 and RAMH (10 mg/kg, i.p) were co-administered with a 15-min interval between the two injections. Treatments were given on all days of the respective behavioral experiments. The Novel Object Recognition Test (NORT) and Fear Conditioning Test (FCT) were conducted over 3 days, while the Y-maze (spontaneous alternation), three-chamber and elevated plus maze tests were conducted as single-day experiments. A three-day gap was maintained between different behavioral studies, during which no treatments were administered. After completing all behavioral tests, mice were sacrificed. The animals were deeply anesthetized with pentobarbital (40 mg/kg, i.p.) to ensure loss of consciousness. Following anesthesia, transcardial perfusion was performed. Brains were then carefully isolated and placed on an ice-cold plate for dissection. For subsequent biochemical analyses, the hippocampus and cerebellum were separated and immediately flash-frozen in liquid nitrogen. Figure 2 depicts the timeline for drug treatments and behavioral studies conducted in SCO treated amnesic mice.

### 2.4 Behavioral assessments

#### 2.4.1 Novel object recognition test

NORT was conducted in an open-field arena to assess memory in mice, according to previously published protocols (Lueptow, 2017; Abdalla et al., 2023). Discrimination index was calculated using the following formula: *[T2—T1]/[T2* + *T1] × 100*, T2 and T1 represent time spent in exploring the novel object and the familiar object respectively.

#### 2.4.2 Elevated plus maze

To assess levels of anxiety, the procedure outlined in our previously published studies was implemented (Bahi, 2013; Eissa et al., 2020b). Total entries into the open arms and the duration spent in the open arms were measured to assess anxiety levels over the 5-min period.

#### 2.4.3 Y-Maze test

To assess Spontaneous alternation: The test protocol for YMT, based on a previous method (Garcia and Esquivel, 2018; Kim et al., 2023) was used to evaluate short-term spatial memory by leveraging rodents’ natural curiosity to explore new environments. The test used a gray acrylic Y-maze with three arms, each labeled A, B, and C. Mice were allowed to explore freely while an experimenter recorded the sequence of arm entries for 8 min. A mouse was considered to have visited an arm when all four limbs crossed an imaginary line on the maze floor. Mice were considered to have made alternations if they visited all three arms sequentially without repetition. Spontaneous alternation (*%*), used as an indicator of spatial memory, was calculated using a specific formula: *Number of alternations/Total arm entries-2* × *100*

#### 2.4.4 Three chamber test

TCT for social interaction and social novelty preference was conducted over three 10-min sessions as per our previously published studies (Eissa et al., 2018; Venkatachalam et al., 2021). The sociability index (SI) and social novelty preference index (SNI) were used to compare social behaviors between the treated groups. The sociability index (SI) was calculated from *[Time exploring NM −Time exploring NO]/[Time exploring NM* + *Time exploring NO]*; while social novelty index (SNI) was calculated from *[Time exploring NM − Time exploring FM]/[Time exploring NM* + *Time exploring FM]*. In this test, “NM” refers to the Novel Mouse, “NO” refers to the Novel Object, and “FM” refers to the Familiar Mouse.

#### 2.4.5 Fear conditioning test

The protocol for assessing associative learning involved three main sessions across 3 days (Shoji et al., 2014; Yu et al., 2023). On the first day (Day 1), mice underwent a conditioning session (120 s) in a square chamber with an electrifiable grid floor. Subsequently, an auditory cue (3,600 Hz tone) is presented as the conditioned stimulus (CS) for 20 s, followed by a 0.5 mA foot-shock as the unconditioned stimulus (US) during the last 2 s of the sound. After a 120-s interval, the pairing of the auditory cue and foot-shock is repeated. Following the last foot-shock, mice are left undisturbed in the chamber for an additional 120 s before being returned to their home cage. On the second day (Day 2), the mice were placed back in the same chamber for a context test, and freezing behavior was measured for 3 min to assess context-dependent fear. The cued test is conducted the following day, which marked Day 3 of the experiment. The cued test took place in a novel chamber with distinct sensory cues, and the previously used auditory cue was presented again to measure freezing behavior in response to the cue in a new context. The percentage of time spent freezing during the sessions on Day 2 and 3 was recorded as an indicator of associative learning.

### 2.5 Biochemical assays

#### 2.5.1 Brain collection and tissue processing

Following the protocol established (Eissa et al., 2019), brain tissues were collected post behavioral studies, processed using RIPA buffer, and stored for analysis of Caspase-1 activity, oxidative stress markers, proinflammatory cytokines and acetylcholinesterase activity.

#### 2.5.2 Caspase-1 activity

Caspase-1 activity was measured using a fluorometric assay kit (Abcam, United Kingdom) targeting the YVAD sequence. Samples were incubated with reaction buffer and YVAD-AFC substrate at 36°C for 1 h, and fluorescence was recorded at 400/505nm. Five mice were used per group were used for the analysis.

#### 2.5.3 Estimation of oxidative stress markers

##### 2.5.3.1 Malondialdehyde

The concentration of malondialdehyde (MDA), a marker of lipid peroxidation, was measured using an MDA detection kit from Northwest Life Science (Vancouver, WA, United States), following the manufacturer’s instructions (Eissa et al., 2018, 2021). Five mice were used per group for the estimation of MDA levels.

##### 2.5.3.2 Glutathione (GSH)

Glutathione levels were quantified using a commercially available GSH kit, adhering to the manufacturer’s protocol (Sigma-Aldrich Chemie GmbH, Steinheim). The results were expressed as μM GSH per mg of protein (Eissa et al., 2018, 2021). Five mice were used per group for the estimation of GSH levels.

##### 2.5.3.3 Antioxidant enzyme, superoxide dismutase

The activity of the endogenous antioxidant enzyme superoxide dismutase (SOD) was assessed using commercially available assay kit from Cayman Chemicals Co. (Ann Arbor, MI, United States). Absorbance was measured at 450 nm using a microplate reader, and SOD activity was expressed in units/mg protein (Eissa et al., 2018, 2021). Five mice were used per group for the estimation of SOD levels.

#### 2.5.4 Pro-inflammatory cytokine assessments

ELISA was used to measure the levels of interleukin-1β (IL-1β) and tumor necrosis factor-alpha (TNF-α), in the cerebellum and hippocampus regions (Thomas et al., 2024b). The assays utilized commercially available ELISA kits (DuoSet) from R&D Systems, and the quantification was conducted according to the manufacturer’s protocol. Results were reported as pg/mg protein. Five mice were used per group for the estimation of pro-inflammatory cytokines.

#### 2.5.5 Assessment of acetylcholinesterase activity in hippocampus and cerebellum

AChE activity was measured following the manufacturer’s instructions (Eissa et al., 2020b). For the estimation of AChE activity, four mice were used per group.

### 2.6 Data analyses

Behavioral test data were presented as means with standard error of the mean (SEM). Compound effects were assessed using a one-way ANOVA, with *post hoc* comparisons conducted via Tukey’s test. For biochemical data, a one-way ANOVA was performed, followed by a Tukey’s multiple comparison test. Statistical analyses were carried out using the Prism GraphPad software, and a *p*-value of less than 0.05 (*p* < 0.05) was considered statistically significant.

## 3 Results

### 3.1 E100 ameliorated SCO-induced short-term memory and long-term memory deficits in C57 mice

**FIGURE 3:**
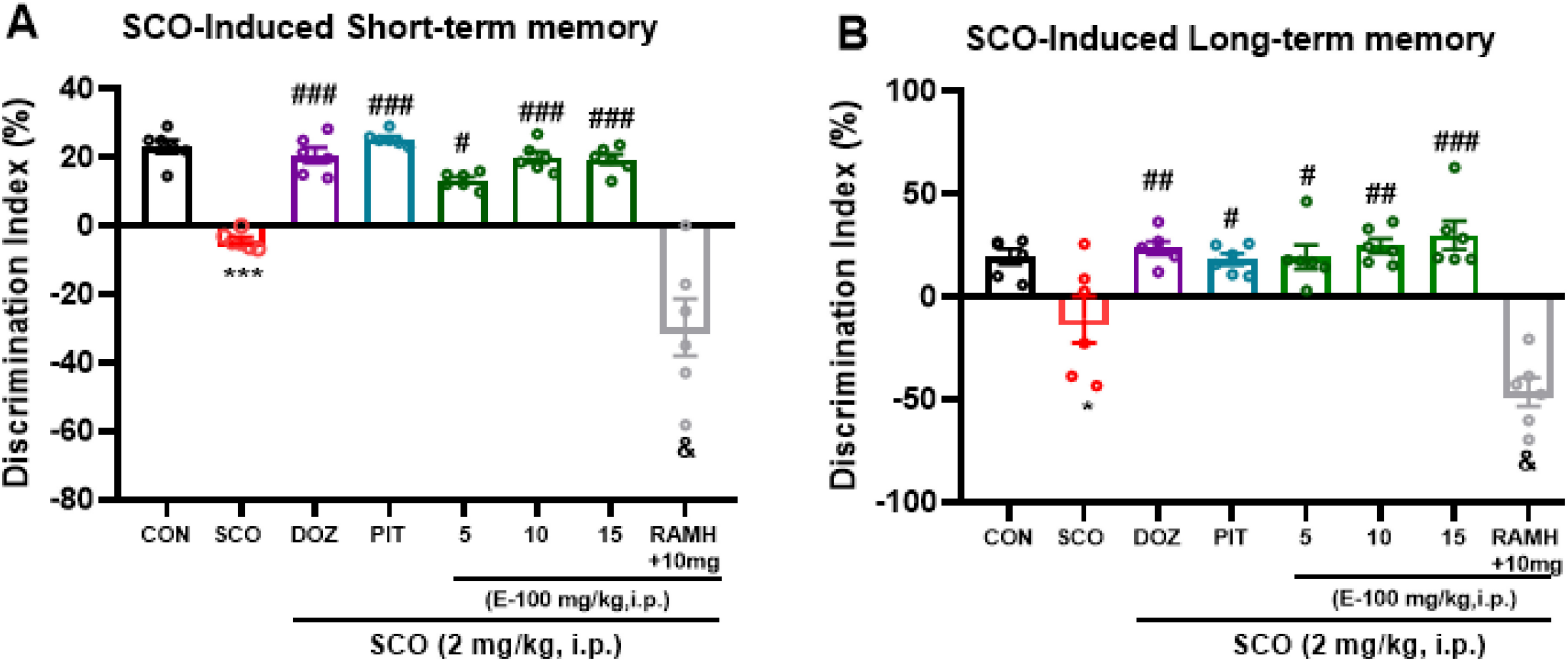
Novel object recognition test: Memory impairment was induced by i.p. injection of scopolamine (2 mg/kg). The control group received normal saline. E100 (5, 10, 15 mg/kg, i.p), DOZ (1 mg/kg, i.p), PIT (10 mg/kg, i.p) and RAMH were injected 15–30 min before each behavioral test. E100 ameliorated SCO-induced short-term and long-term memory deficits in C57 mice. **(A,B)** Represent observed Discrimination Index (DI) in short-term memory (STM) and long-term memory (LTM) tests in SCO treated mice. E100 (5, 10 and 15 mg/kg, i.p.) or DOZ (1 mg/kg, i.p.) or PIT (10 mg/kg, i.p.) improved short-term and long-term memory deficits in SCO-treated mice. The columns represent the means ± SEM (*n* = 6). Statistical analysis was performed using one-way ANOVA followed by Tukey’s multiple comparison test.*p < 0.05, ***p < 0.001 vs. naive control mice. ^#^*p* < 0.05 vs. SCO mice. ^##^*p* < 0.01 vs. SCO mice. ^###^*p* < 0.001 vs. SCO mice. ^&^*p* < 0.001 vs. E100 (10 mg) -treated mice. DI, Discrimination Index; STM, Short-Term Memory; LTM, Long-Term Memory; SCO, scopolamine; DOZ, Donepezil; PIT, Pitolisant; RAMH, (R)-α-Methylhistamine; SEM, standard error of the mean.

Figure 3A illustrates a significant difference in the observed Discrimination Index (DI) in the group pre-treated with SCO (2 mg/kg), showing a *p* < 0.001 compared to the naive control group in STM test [*F*(1, 10) = 152.5, *p* < 0.001]. Similarly, in the LTM test, the group treated with SCO (2 mg/kg) showed a significant decrease in DI compared to the naive control group, with a *p* < 0.05 [*F*(1, 10) = 6.54, *p* < 0.05] (Figure 3B). The observed decline in DI in the SCO-treated group supports the well-established role of SCO as a model for memory impairment, as it disrupts cholinergic neurotransmission—a key mechanism underlying learning and memory processes. These results confirm that SCO administration induces cognitive deficits, particularly affecting the ability to differentiate between novel and familiar objects, as measured by DI. When compared to the SCO-treated amnesic mice, the data reveal a significant increase in DI in the groups treated with DOZ and PIT following systemic administration of SCO (2 mg/kg), in the STM test (Figure 3A, *p* < 0.001 respectively) and the LTM test (Figure 3B, *p* < 0.01 and < 0.05 respectively). Both DOZ, an AChEI, and PIT, a H3R antagonist, were used as positive controls to demonstrate the potential for memory improvement. Both treatments significantly enhanced STM and LTM memory performance, as indicated by the higher DI values. Both compounds are known to enhance cholinergic neurotransmission and modulate histaminergic pathways, leading to improved memory performance in both STM and LTM paradigms.

Furthermore, in the STM test, mice with SCO induced amnesia that received E100 at doses of 10 and 15 mg/kg exhibited a significant rise in DI in comparison with control amnesic mice (*p* < 0.001) {[*F*(1, 10) = 154.6, *p* < 0.001], [*F*(1, 10) = 167.49, *p* < 0.001], respectively}. However, the group treated with a 5 mg/kg showed a lesser significant improvement. There were no significant differences in short-term memory performance between the DOZ-treated group and the E100-treated groups at 10 and 15 mg/kg (all *p* > 0.05). Likewise, short-term memory performance did not differ significantly between the PIT-treated group and the E100-treated groups at the same doses (all *p* > 0.05). Moreover, SCO-treated mice that were administered E100 (at doses of 5, 10, and 15 mg/kg) displayed a substantial increase in DI in the LTM test {[*F*(1, 10) = 5.63, *p* < 0.05], [*F*(1, 10) = 9.15, *p* < 0.01], and [*F*(1, 10) = 10.48, *p* < 0.001] respectively}. The observed dose-dependent improvement in DI suggests that E100 enhances the cognitive performance by modulating neurotransmitter systems involved in memory processes. No significant differences in long-term memory performance were observed between the DOZ-treated group and the E100-treated groups at doses of 5, 10, and 15 mg/kg. Similarly, long-term memory performance did not differ significantly between the PIT-treated group and the E100-treated groups (all *p* > 0.05). In an additional experiment, SCO-treated mice co-injected with the H3R agonist RAMH (10 mg/kg) and E100 (10 mg/kg) exhibited a significant reduction in DI (*p* < 0.001) when compared to SCO-treated mice that received only E100 (10 mg/kg) in both the STM and LTM tests. These findings suggest that H3R activation counteracts the memory-enhancing effects of E100, leading to a reduction in cognitive performance. Since H3R activation is known to inhibit the release of key neurotransmitters such as acetylcholine and histamine, the observed decline in DI following RAMH co-administration indicates that H3R activation may worsen memory impairment.

### 3.2 E100 alleviated anxiety in mice with SCO-induced amnesia in EPM test

**FIGURE 4:**
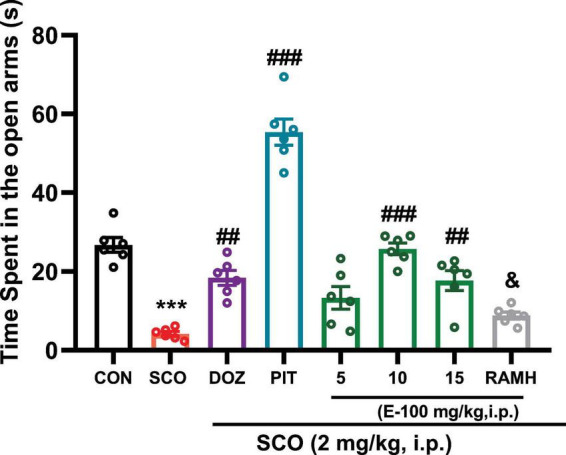
Elevated plus maze test: Memory impairment was induced by i.p. injection of scopolamine (2 mg/kg). The control group received normal saline. E100 (5, 10, 15 mg/kg, i.p), DOZ (1 mg/kg, i.p), PIT (10 mg/kg, i.p) and RAMH were injected 15–30 min before each behavioral test. E100 alleviated anxiety in mice with SCO-induced amnesia in EPM test by increasing the time spent in the open arms in EPM. E100 (5, 10 and 15 mg/kg, i.p.) or DOZ (1 mg/kg, i.p.) or PIT (10 mg/kg, i.p.) increased the time spent in the open arms in SCO-treated mice. The columns represent the means ± SEM (*n* = 6). Statistical analysis was performed using one-way ANOVA followed by Tukey’s multiple comparison test. ***p < 0.001 vs. naive control mice. ^##^*p* < 0.01 vs. SCO mice. ^###^*p* < 0.001 vs. SCO mice. ^&^*p* < 0.001 vs. E100 (10 mg) -treated mice. EPM, Elevated plus maze; SCO, scopolamine; DOZ, Donepezil; PIT, Pitolisant; RAMH, (R)-α-Methylhistamine; SEM, standard error of the mean.

Figure 4 illustrates a significant difference in the time spent by mice in the open arms of the EPM, a well-established test for measuring anxiety in animal models. The data demonstrate that the group treated with SCO (2 mg/kg), a compound known to induce anxiety-like behavior, spent significantly less time in the open arms compared to the naive control group. Statistical analysis revealed a significant difference with a *p* < 0.001 [*F*(1, 10) = 131.91, *p* < 0.001]. The reduced time in the open arms of the SCO-treated group reflects heightened anxiety levels, as spending more time in the open arms generally suggests lower anxiety and greater exploratory behavior. In comparison to the SCO-treated group, the results reveal a marked increase in the time spent in the open arms by mice treated with either DOZ, an AChEI or PIT, a H3R antagonist. Both DOZ and PIT were administered following systemic treatment with SCO (2 mg/kg), and both interventions led to a significant reduction in the anxiety-like behavior induced by SCO. DOZ-treated mice spent a significantly greater amount of time in the open arms, with a *p* < 0.01, suggesting that the AChEI treatment was effective in alleviating SCO-induced anxiety. Similarly, PIT treatment led to an even more pronounced effect, with a *p* < 0.001, suggesting a stronger anxiolytic effect. This comparison highlights the ability of these positive control treatments (DOZ and PIT) to mitigate the anxiety induced by SCO, suggesting that both compounds may counteract the anxiety-like behavior seen in the SCO group. Additionally, the experiment further investigated the potential anxiolytic effects of E100. Mice treated with SCO (2 mg/kg) and E100 at doses of 10 mg/kg and 15 mg/kg spent significantly more time in the open arms compared to the SCO-only group. Statistical analysis showed significant increases in open arm time for both the 10 mg/kg and 15 mg/kg doses of E100. The F-ratios [*F*(1, 10) = 192.51, *p* < 0.001 for the 10 mg/kg dose and *F*(1, 10) = 26.84, *p* < 0.01 for the 15 mg/kg dose] confirmed the statistical significance of these findings. These results indicate that E100 has a dose-dependent anxiolytic effect, with higher doses (10 and 15 mg/kg) significantly reducing the anxiety-like behavior observed in the SCO-treated mice. Notably, the 10 mg/kg dose appeared to be more effective than the 15 mg/kg dose, suggesting an optimal effect at this concentration. In contrast, the 5 mg/kg dose of E100 did not show significant improvement in the time spent in the open arms compared to the SCO-only group (*p* > 0.05). Compared to the DOZ-treated SCO-group, E100 (5, 10, and 15 mg/kg) did not show any significant differences in the time spent in the open arms of the EPM. However, statistical analysis revealed a significant increase in open arm time in the PIT-treated SCO group compared to all doses of E100 (5, 10, and 15 mg/kg) (*p* < 0.001). In a further experiment, SCO-treated mice co-injected with the H3R agonist RAMH and E100 (10 mg/kg) spent significantly less time in the open arms with a *p* < 0.001 compared to those receiving only E100 (10 mg/kg) [*F*(1,10) = 98.55, *p* < 0.001]. This reduction in time spent in the open arms suggests that RAMH, by activating H3Rs, may counteract the anxiolytic effects of E100.

### 3.3 E100 enhanced alternation in mice with SCO-induced amnesia

**FIGURE 5:**
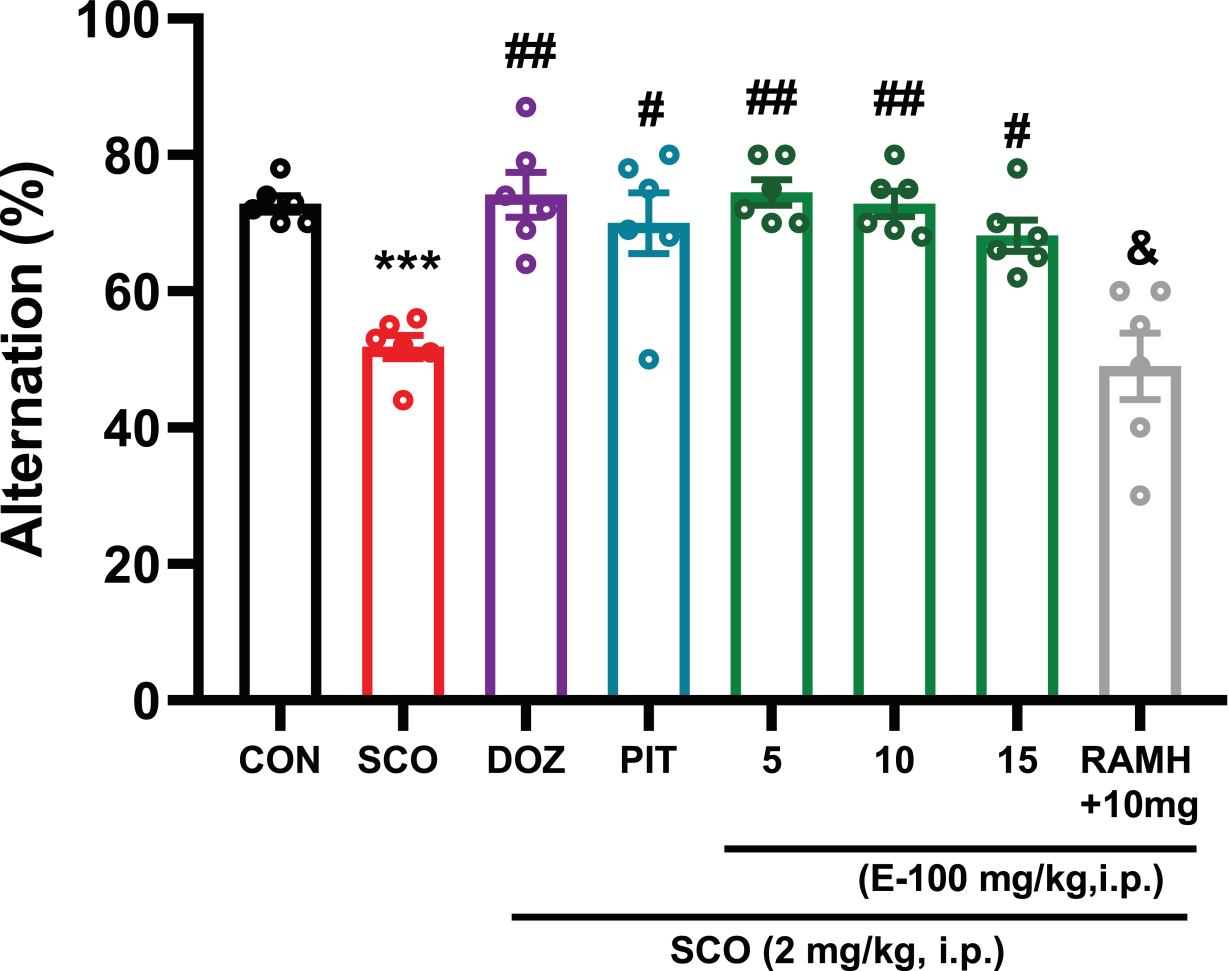
Y-maze test: Memory impairment was induced by i.p. injection of scopolamine (2 mg/kg). The control group received normal saline. E100 (5, 10, 15 mg/kg, i.p), DOZ (1 mg/kg, i.p), PIT (10 mg/kg, i.p) and RAMH were injected 15–30 min before each behavioral test. E100 treatment enhanced the alternation behavior in amnesic mice. The columns represent the means ± SEM (*n* = 6). E100 (5, 10 and 15 mg/kg, i.p) or DOZ (1 mg/kg, i.p) or PIT (10 mg/kg, i.p) considerably improved the alternation in SCO-treated mice. Statistical analysis was performed using one-way ANOVA followed by Tukey’s multiple comparison test. ***p < 0.001 vs. naive control mice. ^#^*p* < 0.01 vs. SCO mice. ^##^*p* < 0.001 vs. SCO mice. ^&^*p* < 0.001 vs. E100 (10 mg)-treated mice. SCO, scopolamine; DOZ, Donepezil; PIT, Pitolisant; RAMH, (R)-α-Methylhistamine; SEM, standard error of the mean.

Statistical analyses (Figure 5) showed a substantial difference in the percentage of alternation in the Y-maze task *(p* < 0.001), reflecting working memory performance, in the group treated with SCO (2 mg/kg), compared to the naive control group [*F*(1, 10) = 97.51, *p* < 0.001]. The reduced percentage of alternation in the SCO-treated group indicates impaired working memory, confirming that SCO induced memory deficits.

When compared to the SCO-treated group, the data exhibit a significant increase in the percentage of alternation in the groups that received systemic administration of DOZ and PIT alongside SCO (2 mg/kg) (*p* < 0.001 and *p* < 0.01, respectively for both groups). Both DOZ and PIT, used as positive controls, demonstrated a substantial capacity to improve working memory, as indicated by their significant impact on memory performance. Specifically, both treatments led to a notable increase in the percentage of alternation underscoring their effectiveness in enhancing cognitive function and memory performance in these mice. Additionally, SCO-treated mice that received E100 at doses of 5, 10 and 15 mg/kg also exhibited improvements in the percentage of alternation, highlighting the memory-enhancing potential of E100. At the lower doses of 5 mg/kg and 10 mg/kg, the increase in alternation was highly significant, with *p* < 0.001 for both doses. At the highest dose of 15 mg/kg, a lesser yet significant improvement was observed, with a *p* < 0.01. These effects were further supported by the corresponding F-values from the statistical analysis: *F*(1, 10) = 77.71 (*p* < 0.001) for the 5 mg/kg dose, *F*(1, 10) = 66.93 (*p* < 0.001) for the 10 mg/kg dose and *F*(1, 10) = 32.84 (*p* < 0.01) for the 15 mg/kg dose. These results highlight the memory-enhancing effects of E100 as evidenced by the increased alternation in Figure 5. There were no significant differences in the percentage of alternation among SCO-induced amnesic mice treated with DOZ, PIT, or E100 indicating comparable efficacy in improving working memory across these treatments (*p* > 0.05). In a further experiment, SCO-treated mice co-injected with the H3R agonist RAMH (10 mg/kg) and E100 (10 mg/kg) showed a significant decrease in the percentage of alternation [*F*(1, 10) = 20.62, *p* < 0.001], compared to those receiving only E100 (10 mg/kg). This reduction in alternation suggests that RAMH, by activating H3Rs, may counteract the working memory-enhancing effects of E100, highlighting a potential inhibitory role of H3R activation on working memory.

### 3.4 E100 enhanced sociability and social novelty index of SCO-treated mice in the three-chamber test

**FIGURE 6:**
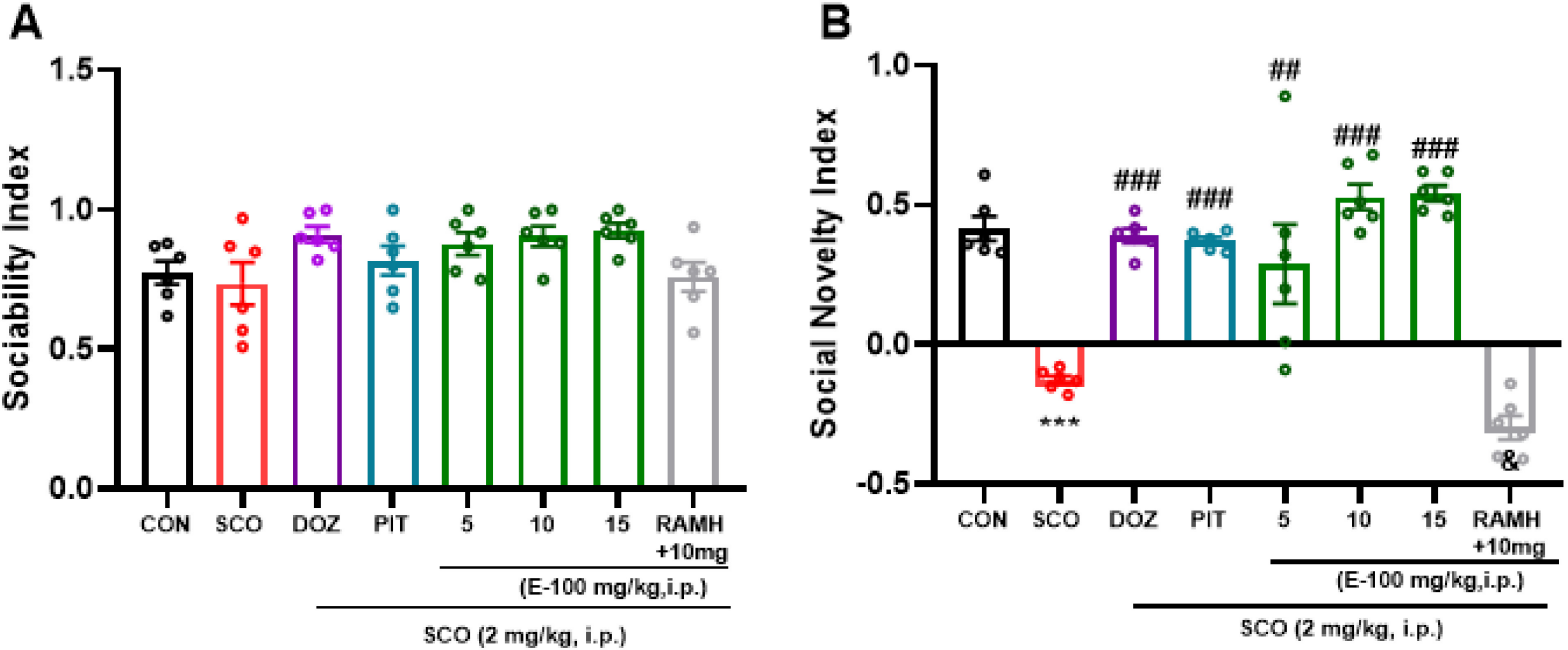
Three chamber test: Memory impairment was induced by i.p. injection of scopolamine (2 mg/kg). The control group received normal saline. E100 (5, 10, 15 mg/kg, i.p), DOZ (1 mg/kg, i.p), PIT (10 mg/kg, i.p) and RAMH were injected 15–30 min before each behavioral test. E100-improved sociability **(A)** and social novelty index **(B)** in SCO-treated mice. E100 (10 and 15 mg/kg, i.p.) or DOZ (1 mg/kg, i.p.) or PIT (10 mg/kg, i.p.) improved the SNI in SCO-treated mice. The columns represent the means ± SEM (*n* = 6). Statistical analysis was performed using one-way ANOVA followed by Tukey’s multiple comparison test. ***p < 0.001 vs. naive control mice. ^##^*p* < 0.05,^###^*p* < 0.001 vs. SCO mice. ^&^*p* < 0.001 vs. E100 (10 mg)-treated mice. SCO, scopolamine; DOZ, Donepezil; PIT, Pitolisant; RAMH, (R)-α-Methylhistamine; SNI, social novelty index; SEM, standard error of the mean.

In the sociability test (Figure 6A), all mouse groups exhibited a high sociability index, demonstrating a clear preference for the compartment containing Stranger 1 over both the empty compartment and the center. This behavior was consistent across all groups, including those with and without induced cognitive impairment, confirming the inherent social nature of mice. These results suggest that cognitive impairment did not affect the basic sociability of the animals. However, in the Social Novelty test (Figure 6B), the SCO-treated group showed a significant decrease in social novelty index (*p* < 0.001), compared to the naive group, indicating that while SCO did not affect sociability, it impaired the mice’s social memory to recognize and differentiate between a familiar and a novel conspecific [*F*(1, 10) = 134.86, *p* < 0.001]. When compared to the SCO-treated amnesic mice (Figure 6B), the data reveal a significant increase in social novelty index in the groups treated with DOZ, an AChEI, and PIT, a H3R antagonist, following systemic administration of SCO (2 mg/kg), indicating that both standard drugs were able to improve the impaired social memory (*p* < 0.001 for both the groups). The observed restoration of social novelty preference suggests that enhancing cholinergic neurotransmission via AChEI (DOZ) or modulating histaminergic signaling via H3R antagonism (PIT) can counteract the impairments caused by scopolamine administration. Furthermore, SCO-treated mice that received E100 at doses of 10 mg/kg and 15 mg/kg also demonstrated a significant improvement in the Social Novelty Index, with *p* < 0.001 {[*F*(1, 10) = 186.48, *p* < 0.001], [*F*(1, 10) = 352.4, *p* < 0.001], respectively}. These results highlight the potential of E100 in improving social memory deficits. In contrast, the improvement observed in the group treated with a 5 mg/kg dose was less significant in SCO-treated mice [*F*(1, 10) = 8.46 *p* < 0.05] as compared to the higher doses. This suggests a dose-dependent efficacy of E100, where higher doses (10 mg/kg and 15 mg/kg) are required to achieve a restoration of social memory. Although the magnitude of improvement observed with E100 (10 and 15 mg/kg) is greater than that seen with DOZ and PIT in amnesic mice, the differences among these groups were not statistically significant (all *p*’s > 0.05). In a subsequent experiment, SCO-treated mice co-injected with the H3R agonist RAMH (10 mg/kg) and E100 (10 mg/kg) exhibited a significant decrease in the percentage of Social Novelty Index (*p* < 0.001), compared to those receiving only E100 (10 mg/kg) [*F*(1, 10) = 175.9, *p* < 0.001]. This reduction in the Social Novelty Index suggests that RAMH, through H3R activation, may counteract the social memory-enhancing effects of E100, indicating a potential inhibitory role of H3R activation on social memory (Figure 6B).

### 3.5 E100 improved freezing in both contextual and cued fear conditioning test in mice with SCO-induced amnesia

**FIGURE 7:**
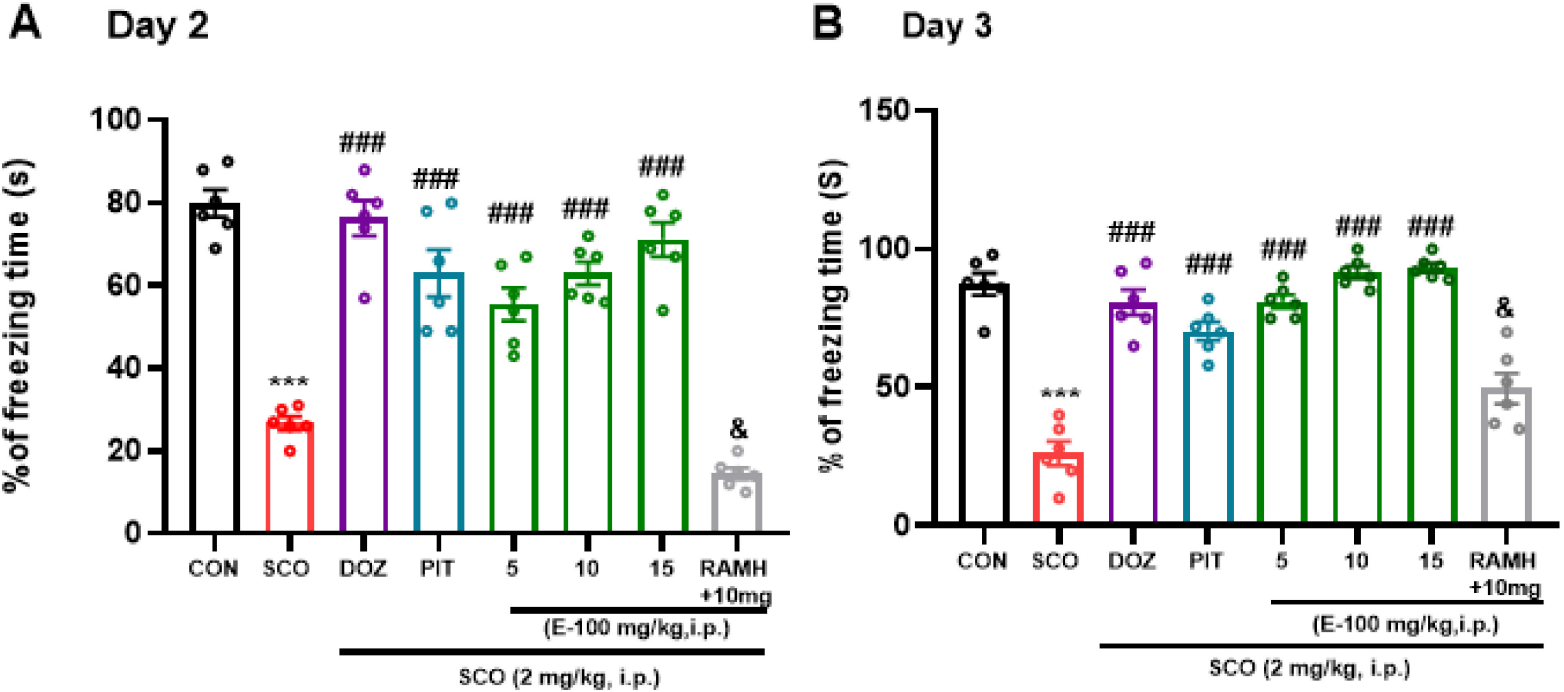
Fear conditioning test: Memory impairment was induced by i.p. injection of scopolamine (2 mg/kg). The control group received normal saline. E100 (5, 10, 15 mg/kg, i.p), DOZ (1 mg/kg, i.p), PIT (10 mg/kg, i.p) and RAMH were injected 15–30 min before each behavioral test. E100 increased the freezing time in both Contextual and cued fear conditioning, **(A)** Percent of freezing in contextual fear test (Day 2) and **(B)** % of freezing in cued fear test (Day 3). The columns represent the means ± SEM (*n* = 6). A significantly lower freezing behavior was seen in amnesic mice compared to naive control group. However, E100 (5, 10 and 15 mg/kg, i.p.) or DOZ (1 mg/kg, i.p.) or PIT (10 mg/kg, i.p.) considerably improved the freezing in SCO-treated mice. Statistical analysis was performed using one-way ANOVA followed by Tukey’s multiple comparison test. ***p < 0.001 vs. naive control mice. ^###^*p* < 0.001 vs. SCO mice. ^&^*p* < 0.001 vs. E100 (10 mg) -treated mice. SCO, scopolamine; DOZ, Donepezil; PIT, Pitolisant; RAMH, (R)-α-Methylhistamine; SEM, standard error of the mean.

The data analyses demonstrate a significant decrease in the percentage of freezing behavior (*p* < 0.001), a well-established indicator of fear memory recall, during both the contextual (Day 2) and cued fear (Day 3) tests in the group treated with SCO (2 mg/kg), compared to the naive control group presented in Figures 7A,B{[*F*(1, 10) = 215.60, *p* < 0.001], [*F*(1, 10) = 106.67, *p* < 0.001], respectively}. This difference in freezing behavior indicates that SCO administration led to substantial memory impairments. In the contextual fear test, as shown in Figure 7A as compared to the SCO-treated group, the results show a significant increase in freezing behavior in the amnesic mice (pre-treated with SCO, 2 mg/kg) administered DOZ and PIT (*p* < 0.001).

Similarly, in the cued fear test illustrated in Figure 7B, both groups exhibited a significant increase in freezing (*p* < 0.001 for both) behavior. Furthermore, SCO-treated mice that were also administered E100 at doses of 5 mg/kg, 10 mg/kg, and 15 mg/kg demonstrated a marked and statistically significant increase in freezing behavior across both the contextual (Figure 7A) and cued fear conditioning tests (Figure 7B) conducted on Days 2 and 3 of the experiment. In the contextual fear test, the increase in freezing behavior was notably significant at all three doses {[*F*(1, 10) = 45.11, *p* < 0.001], [*F*(1, 10) = 128.86, *p* < 0.001], and [*F*(1, 10) = 100.66, *p* < 0.001] for 5, 10, and 15 mg/kg respectively}. Similarly, in the cued fear test conducted on Day 3, all doses of E100 (5, 10, and 15 mg/kg) resulted in a substantial increase in freezing behavior, with a highly significant *p* < 0.001 across all conditions {[*F*(1,10) = 121.3, *p* < 0.001], [*F*(1, 10) = 179.2, *p* < 0.001], and [*F*(1, 10) = 206.68, *p* < 0.001], respectively}. These findings suggest that the administration of E100, in mice pre-treated with SCO, led to a clear enhancement of freezing behavior, reflecting heightened fear responses in both the contextual and cued conditions. Statistical analyses for Day 2 (contextual fear test) indicated no significant differences in freezing behavior between the groups treated with E100 (5, 10 and 15 mg/kg) and DOZ. This finding suggests that the fear memory-enhancing effects of higher doses of E100 are comparable to those produced by DOZ. On Day 3 (cued fear test), although E100 at 10 and 15 mg/kg produced greater enhancements in freezing behavior than DOZ or PIT, statistical analyses did not reveal significant differences between these groups. In a follow-up experiment, SCO-treated mice co-injected with the H3R agonist RAMH (10 mg/kg) and E100 (10 mg/kg) showed a significant reduction in the percentage of freezing behavior (*p* < 0.001) in both the contextual [*F*(1, 10) = 242.08, *p* < 0.001], and cued fear tests [*F*(1, 10) = 49.24, *p* < 0.001], compared to those treated with E100 (10 mg/kg).

### 3.6 E100 decreased the caspase-1 activity in hippocampus and cerebellum of mice with SCO-induced amnesia

**FIGURE 8:**
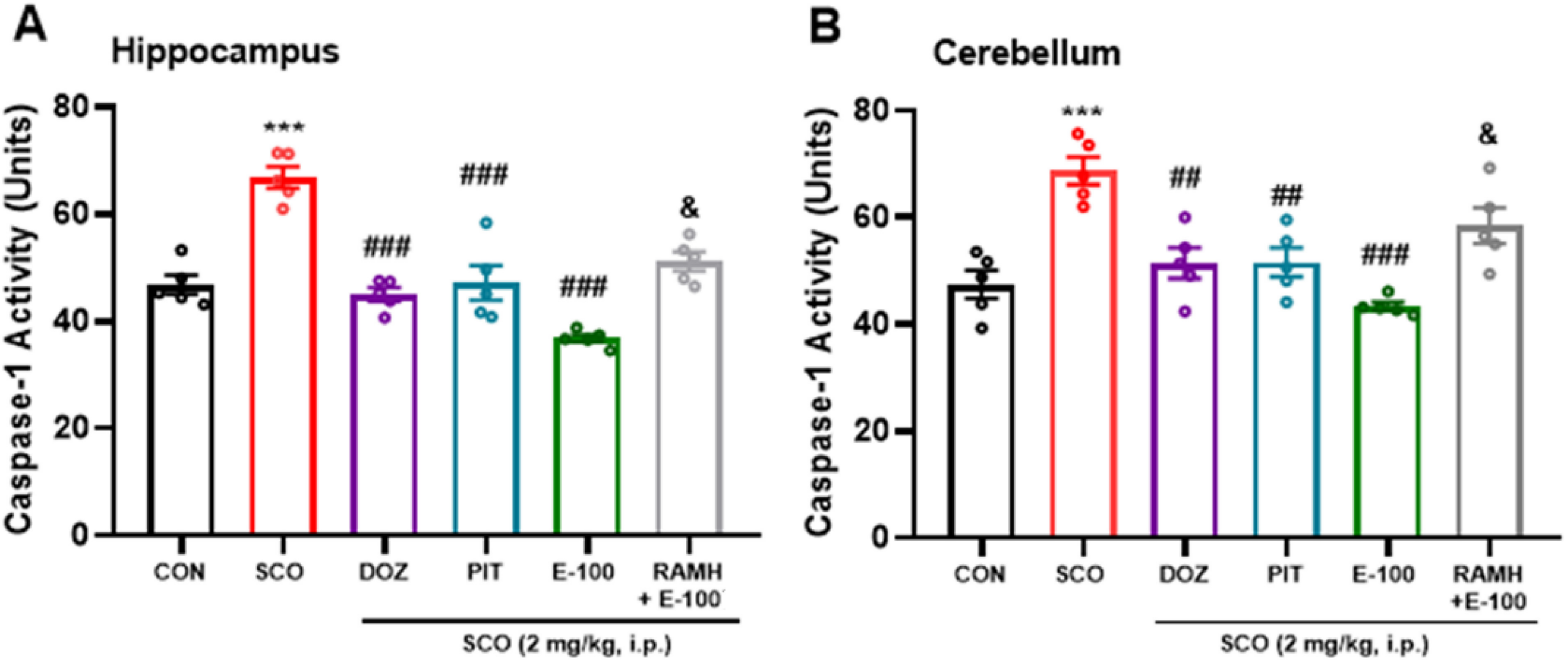
E100 reduced the Caspase-1 activity in hippocampus and cerebellum of amnesic mice. **(A)** Caspase-1 activity in hippocampus, **(B)** Caspase-1 activity in cerebellum. E100 (10 mg/kg, i.p.) or DOZ (1 mg/kg, i.p.) or PIT (10 mg/kg, i.p.) decreased the Caspase-1 activity in SCO-treated mice. The columns represent the means ± SEM (*n* = 5). Statistical analysis was performed using one-way ANOVA followed by Tukey’s multiple comparison test. ***p < 0.001 vs. naive control mice. ^##^*p* < 0.01 vs. SCO mice. ^###^*p* < 0.001 vs. SCO mice.). ^&^*p* < 0.01 vs. E100 (10 mg) -treated mice. SCO, scopolamine; DOZ, Donepezil; PIT, Pitolisant; RAMH, (R)-α-Methylhistamine; SEM, standard error of the mean.

In the SCO-treated group, Figures 8A,B show a marked increase in caspase-1 activity in both the hippocampus and cerebellum, with high significant *p* < 0.001 for both groups, compared to the naive control group [*F*(1, 8) = 54.73, *p* < 0.001] and [*F*(1, 8) = 32.9, *p* < 0.001] for hippocampus and cerebellum respectively. This elevation suggests that scopolamine administration triggers a pronounced neuroinflammatory response, which may contribute to cognitive deficits. In comparison to the SCO-treated group, the data indicate a significant reduction in caspase-1 activity in the DOZ and PIT-treated groups after systemic administration of Scopolamine (2 mg/kg). In the hippocampus, both groups showed a significant decrease with a *p* < 0.001. Likewise, in the cerebellum, both groups demonstrated a significant reduction (*p* < 0.01 for both groups). These results suggest that enhancing cholinergic transmission via DOZ and modulating histaminergic signaling via PIT may attenuate neuroinflammation. Interestingly, SCO-treated mice that received E100 (10 mg/kg) exhibited a significant decrease in the caspase-1 activity in both hippocampus and cerebellum [*F*(1, 8) = 163.36, *p* < 0.001] and [*F*(1, 8) = 86.74, *p* < 0.001] for hippocampus and cerebellum respectively. By mitigating caspase-1 activation, E100 reduces the neuroinflammatory burden associated with scopolamine-induced cognitive impairment. While the reduction in caspase-1 levels was more pronounced with E100 treatment in hippocampus and cerebellum compared to DOZ and PIT, statistical analyses revealed no significant differences between these groups. In an additional experiment, SCO-treated mice co-injected with the H3R agonist RAMH (10 mg/kg) and E100 (10 mg/kg) showed a significant increase in the caspase-1 activity, with *p* < 0.01 in both the hippocampus [*F*(1, 8) = 57.48, *p* < 0.01] and cerebellum [*F*(1, 8) = 19.21, *p* < 0.01], compared to the E100 (10 mg/kg) treated group. This increase suggests that H3R activation via RAMH may counteract the anti-inflammatory effects of E100, potentially exacerbating neuroinflammation.

### 3.7 E100 decreased oxidative stress in hippocampus and cerebellum of mice with SCO-induced amnesia

**FIGURE 9:**
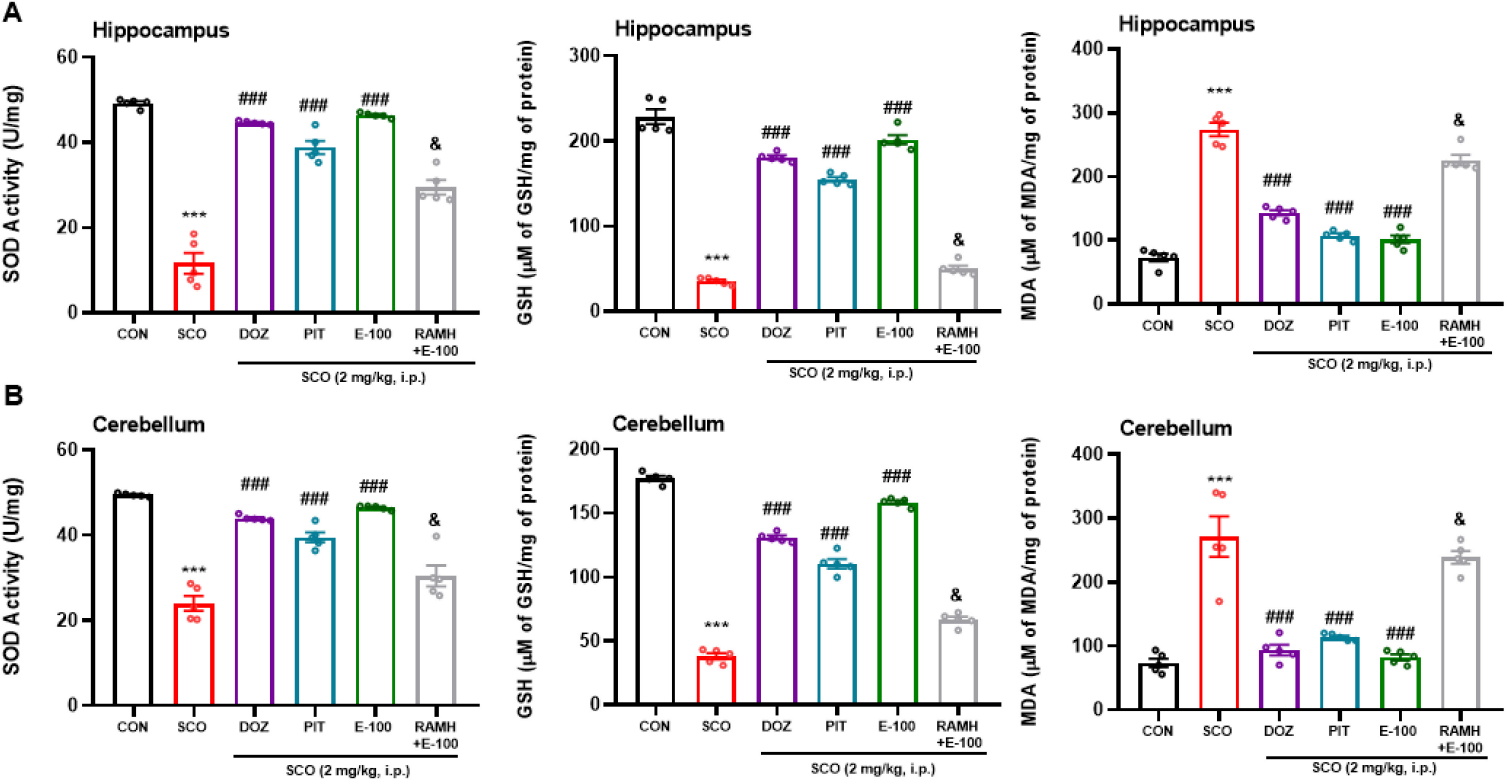
E100 mitigated oxidative stress in amnesic C57 mice. The oxidative stress markers Malondialdehyde (MDA, μM MDA/mg of protein), Superoxide dismutase (SOD, units/mg protein), and Glutathione (GSH, μM GSH/mg protein) levels were measured in hippocampus **(A)** and cerebellum **(B)** of mice with SCO-induced amnesia. Treatment with E100 (10 mg/kg, i.p.) or DOZ (1 mg/kg, i.p.) or PIT (10 mg/kg, i.p.) increased GSH and SOD and reduced MDA levels in amnesic mice. The columns represent the means ± SEM (*n* = 5). (Statistical analysis was performed using one-way ANOVA followed by Tukey’s multiple comparison test) ***p < 0.001 vs. naive mice. ^###^*p* < 0.001 vs. SCO mice. ^&^*p* < 0.001 vs. E100 (10 mg) -treated mice. SCO, scopolamine; DOZ, Donepezil; PIT, Pitolisant; RAMH, (R)-α-Methylhistamine; SEM, standard error of the mean.

In the SCO-treated group, a marked dysregulation in oxidative stress markers was observed. Specifically, there was a significant reduction in the levels of superoxide dismutase (SOD) and glutathione (GSH), two crucial antioxidants responsible for maintaining redox homeostasis. Concurrently, a notable increase in malondialdehyde (MDA) activity was detected in both the hippocampus (Figure 9A) and cerebellum (Figure 9B) when compared to the naïve control group. The statistical significance was extremely high, with *p* < 0.001 when compared to the naive control group, indicating a substantial impairment in the brain’s antioxidant defense mechanisms in SCO treated group {[*F*(1, 8) = 232.23, *p* < 0.001] and [*F*(1, 8) = 207.1, *p* < 0.001] in hippocampus and cerebellum respectively for SOD and [*F*(1, 8) = 473.27, *p* < 0.001] and [*F*(1, 8) = 199.17, *p* < 0.001] in hippocampus and cerebellum respectively for GSH}.

The increased levels of MDA, a marker of lipid peroxidation, further confirm the extent of oxidative damage, which may contribute to neuronal dysfunction and cognitive decline in SCO-treated animals {[*F*(1, 8) = 276.04, *p* < 0.001] and [*F*(1, 8) = 37.47, *p* < 0.001] in hippocampus and cerebellum}. Compared to the SCO-treated group, systemic administration of DOZ, PIT, and E100 (10 mg/kg) resulted in a significant increase in SOD {[*F*(1, 8) = 203.14, *p* < 0.001] and [*F*(1, 8) = 160.88, *p* < 0.001] in hippocampus and cerebellum respectively for E100 administered group as compared to SCO treated group} and GSH levels {[*F*(1, 8) = 853.8, *p* < 0.001] and [*F*(1, 8) = 847.8, *p* < 0.001] in hippocampus and cerebellum respectively for E100 administered group in comparison to SCO treated group} and a substantial reduction in MDA in both the hippocampus {[*F*(1, 8) = 203.6, *p* < 0.001] for E100 administered group as compared to SCO treated group} (Figure 9A) and [*F*(1, 8) = 35.59, *p* < 0.001] cerebellum (Figure 9B). These protective effects were highly significant (*p* < 0.001) across all treatment groups, suggesting that these compounds effectively counteract SCO-induced oxidative stress. When comparing antioxidant effects among treatments, no statistically significant differences were found in SOD levels between the DOZ and E100 (10 mg/kg) groups in either brain region, however, significant differences were observed between the PIT and E100 treated groups in both the hippocampus and cerebellum (*p* < 0.05). Similarly, GSH levels were also significantly different between the PIT and E100 treated groups (*p* < 0.01), with E100 showing a more pronounced restoration of glutathione levels in both brain regions. With respect to MDA levels, E100 (10 mg/kg) treatment showed a significantly greater reduction in lipid peroxidation in hippocampus compared to DOZ-treated mice (*p* < 0.01), highlighting its superior efficacy in mitigating oxidative damage. However, no statistically significant difference in MDA levels was observed between the E100 and PIT treatment groups. To further explore the involvement of H3 receptors, an additional experiment was conducted in which SCO-treated mice were co-injected with RAMH (10 mg/kg) and E100 (10 mg/kg). The findings revealed that the combination of RAMH with E100 led to a significant decrease in SOD and GSH levels, {[*F*(1, 8) = 99.81, *p* < 0.001] and [*F*(1, 8) = 42.03, *p* < 0.001] in hippocampus and cerebellum respectively for SOD as well as [*F*(1, 8) = 545.60, *p* < 0.001] and [*F*(1, 8) = 694.66, *p* < 0.001] in hippocampus and cerebellum respectively for GSH}, along with an increase in MDA {[*F*(1, 8) = 203.68, *p* < 0.001] in hippocampus and [*F*(1, 8) = 35.5, *p* < 0.001] in cerebellum} activity. These changes were highly significant (*p* < 0.001) when compared to the E100 (10 mg/kg) treated group, suggesting that activation of H3R counteracts the antioxidative effects of E100.

### 3.8 E100 decreased the level of proinflammatory cytokines in hippocampus and cerebellum of mice with SCO-induced amnesia

**FIGURE 10:**
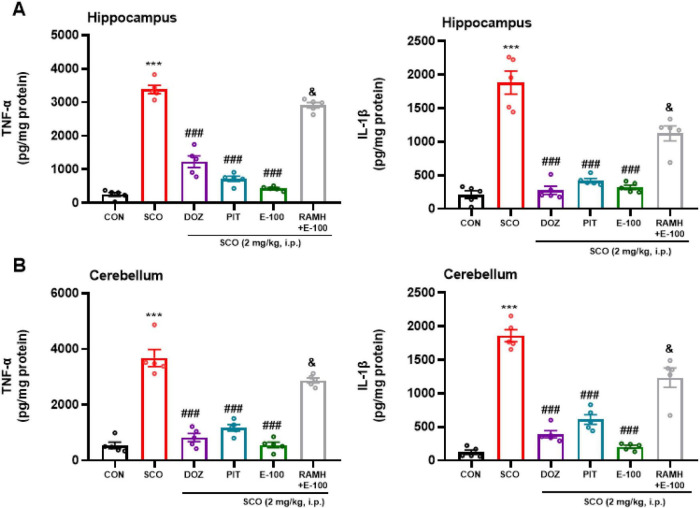
E100 mitigated neuroinflammation in hippocampus **(A)** and cerebellum **(B)** of SCO-induced amnesic mice. Tumor Necrosis Factor (TNF-α) and interleukin (IL-1β) were assessed. SCO-induced amnesic mice exhibited considerable elevation in tested cytokine levels in hippocampus and cerebellum compared to control mice. E100 (10 mg/kg, i.p.) or DOZ (1 mg/kg, i.p.) or PIT (10 mg/kg, i.p.) significantly decreased TNF-α and IL-1β levels in cerebellum and hippocampus. The columns represent the means ± SEM (*n* = 5). (Statistical analysis was performed using one-way ANOVA followed by Tukey’s multiple comparison test) ***p < 0.001 vs. naive mice. ^###^*p* < 0.001 vs. SCO mice. ^&^*p* < 0.001 vs. E100 (10 mg) -treated mice. SCO, scopolamine; DOZ, Donepezil; PIT, Pitolisant; RAMH, (R)-α-Methylhistamine; SEM, standard error of the mean.

As demonstrated in Figure 10, systemic administration of SCO (2 mg/kg) led to a significant elevation in the levels of pro-inflammatory cytokines, tumor necrosis factor-alpha (TNF-α) and interleukin-1 beta (IL-1β) in both the hippocampus (Figure 10A) and cerebellum (Figure 10B) when compared to the naïve control group (*p* < 0.001) {[*F*(1, 8) = 505.03, *p* < 0.001] and [*F*(1, 8) = 91.14, *p* < 0.001] in hippocampus and cerebellum respectively for TNF-α as well as [*F*(1, 8) = 84.70, *p* < 0.001] and [*F*(1, 8) = 327.10, *p* < 0.001] in hippocampus and cerebellum respectively for IL-1β}. This suggests that SCO triggers a pronounced neuroinflammatory response in these brain regions, which may contribute to the cognitive impairments typically associated with SCO administration.

To evaluate the potential therapeutic effects of DOZ, PIT, and E100, these treatment groups were administered following SCO exposure. Notably, all three treatment groups as seen in Figure 10 exhibited a significant reduction in TNF-α and IL-1β levels in both the hippocampus and cerebellum (*p* < 0.001). E100 exhibited a significant decrease in TNF-α and IL-1β levels in both the tested regions as compared the scopolamine treated group {[*F*(1, 8) = 521.06, *p* < 0.001] and [*F*(1, 8) = 93.31, *p* < 0.001] in hippocampus and cerebellum respectively for TNF-α} as well as {[*F*(1, 8) = 84.7, *p* < 0.001] and [*F*(1, 8) = 327.4, *p* < 0.001] in hippocampus and cerebellum respectively for IL-1β}. This highlights the robust anti-inflammatory effects of these treatments in counteracting SCO-induced neuroinflammation. The observed reduction in cytokine levels suggests that DOZ, PIT, and E100 might mitigate neuroinflammatory pathways, potentially alleviating cognitive and neurodegenerative impairments associated with SCO administration. Further analysis revealed that in the cerebellum, IL-1β was significantly lower in the E100-treated group compared to the PIT group (*p* < 0.05), indicating a stronger anti-inflammatory effect of E100 in this region. However, no significant difference was observed in the cerebellum between the E100 and DOZ groups. With respect to TNF-α, the E100 group showed significantly lower levels compared to the DOZ-treated group in the hippocampus (*p* < 0.01), suggesting superior efficacy of E100 in reducing hippocampal TNF-α levels. In an additional experiment, mice treated with SCO and co-injected with the H3R agonist RAMH (10 mg/kg) and E100 (10 mg/kg) demonstrated a marked increase in TNF-alpha and IL-1beta levels, in both brain regions, compared to the group treated with E100 (10 mg/kg) alone (*p* < 0.001) {[*F*(1, 8) = 802.8, *p* < 0.001] and [*F*(1, 8) = 291.5, *p* < 0.001] in hippocampus and cerebellum respectively for TNF-α} as well as {[*F*(1, 8) = 47.2, *p* < 0.001] and [*F*(1, 8) = 49.4, *p* < 0.001] in hippocampus and cerebellum respectively for IL-1β}.

### 3.9 E100 decreased the level of acetylcholine esterase in hippocampus and cerebellum of mice with SCO-induced amnesia

**FIGURE 11:**
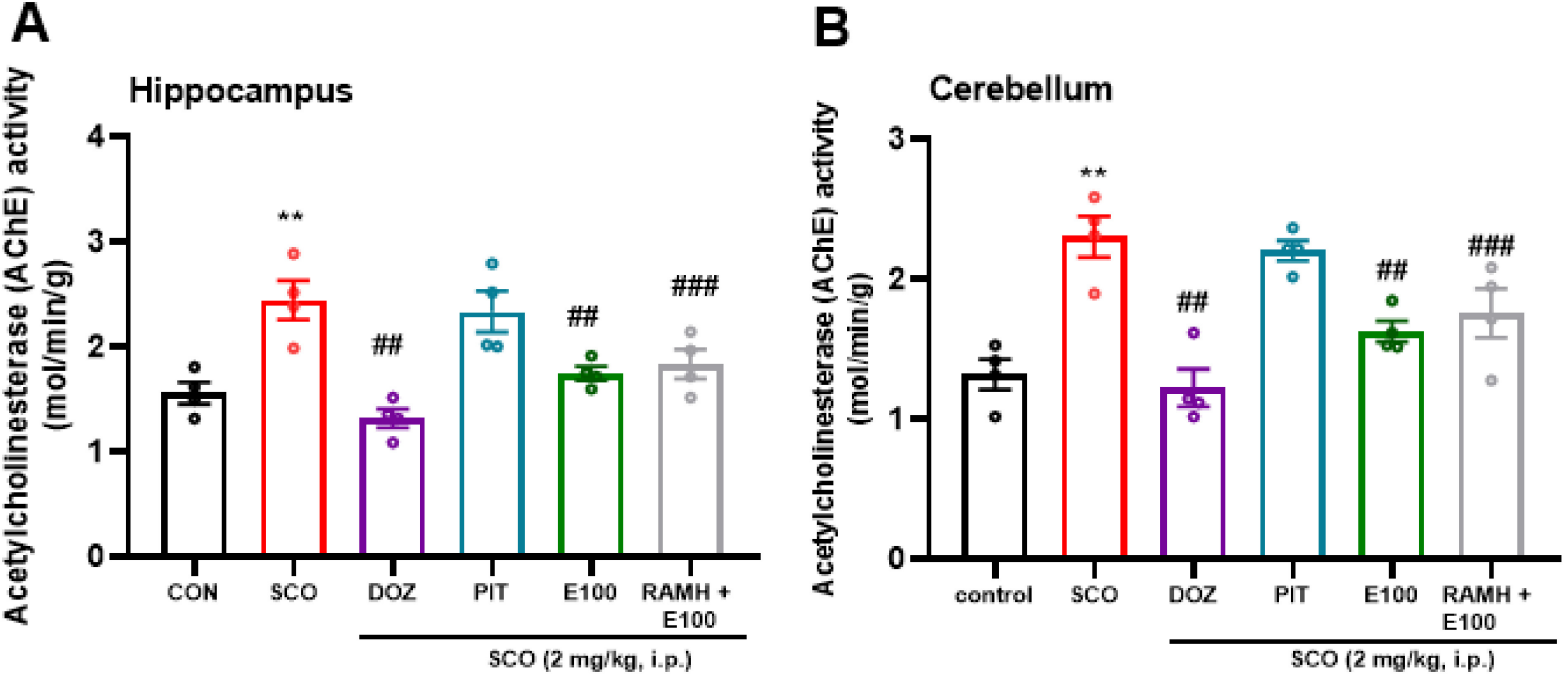
E100 (10 mg/kg, i.p.) reduced the enhanced AChE activity levels in the hippocampus **(A)** and cerebellum **(B)** of SCO-induced amnesic mice. Statistical analysis revealed a significant increase in the AChE activity in hippocampus and cerebellum of SCO-induced amnesic mice compared to the control mice. E100 (10 mg/kg, i.p.) or DOZ (1 mg/kg, i.p.) pre-treatment significantly reduced this activity in amnesic mice. The columns represent the means ± SEM (*n* = 4). (Statistical analysis was performed using one-way ANOVA followed by Tukey’s multiple comparison test) **p < 0.01 vs. naive control mice. **^##^***p* < 0.01 vs. SCO mice. **^###^***p* < 0.05 vs. SCO mice. SCO, scopolamine; DOZ, Donepezil; PIT, Pitolisant; RAMH, (R)-α-Methylhistamine; SEM, standard error of the mean; AChE, Acetylcholinesterase.

Acetylcholine esterase (AChE) activity in SCO-induced amnesic mice was assessed in the hippocampus and cerebellum (Figure 11). The results demonstrated that amnesic mice exhibited significant increase in the activity of acetylcholine esterase enzyme in the hippocampal (Figure 11A) and cerebellar tissues (Figure 11B) compared to the naive control group [*F*(1, 6) = 17.37, *p* < 0.01] and [*F*(1, 6) = 28.9, *p* < 0.01] for hippocampus and cerebellum respectively indicating cholinergic dysfunction associated with memory impairment. The AChE activity of SCO-induced amnesic mice was observed to be significantly decreased in the hippocampus and cerebellum following pretreatment with DOZ (1 mg/kg.), a well-established AChE inhibitor when compared with the SCO-induced amnesic mice (*p* < 0.01) (Figure 11). Pretreatment with PIT failed to produce any significant reduction in AChE activity in either the hippocampus or cerebellum relative to the SCO group (*p* > 0.05), suggesting a lack of efficacy in modulating cholinergic enzyme activity. Furthermore, acute treatment with E100 (10 mg/kg, i.p.) showed a significant decrease in the AChE activity in hippocampal and cerebellar tissues of SCO-treated mice in comparison to the SCO-induced amnesic mice (*p* < 0.01) [*F*(1, 6) = 12.61, *p* < 0.01] and [*F*(1, 6) = 16.72, *p* < 0.01] for hippocampus and cerebellum respectively. Notably, no significant difference was found between E100 and DOZ treated amnesic mice in attenuating AChE activity in both the hippocampus and cerebellum (all *p* > 0.05). Furthermore, E100 was also significantly more effective than PIT in reducing AChE activity (*p* < 0.05). Moreover, co-administration of E100 (10 mg/kg) and RAMH (10 mg/kg) resulted in AChE activity levels that were not significantly different from the E100 group (*p* > 0.05), indicating that RAMH did not abrogate the E100 mediated inhibition of AChE activity. However, co-administration of E100 and RAMH exhibited a significant reduction in AChE activity compared to the SCO-induced amnesic mice (*p* < 0.05), further supporting the efficacy of E100 in mitigating SCO-induced cholinergic dysfunction.

## 4 Discussion

Cognitive decline has been linked to changes in multiple neurotransmitter levels, oxidative brain damage, and alterations in brain structure (Haam and Yakel, 2017). The cholinergic hypothesis, proposed by Bartus et al. (1982), highlights the degeneration of cholinergic neurons and the resulting ACh deficiency as a key factor in AD-related memory loss (Bartus et al., 1982; Hampel et al., 2019). Cholinesterase inhibitors (CHEIs) help maintain ACh levels by preventing its breakdown, thereby supporting cholinergic function and slowing cognitive decline (Tang, 2019; Eshaghi Ghalibaf et al., 2023). ChEIs may also work through alternative mechanisms, helping to reduce free radicals, mitigate amyloid toxicity, and suppress cytokine release from activated microglia in the brain (Benfante et al., 2021).

The H3R which is primarily and heterogeneously expressed in the CNS, plays a significant role in cognitive functions (Sadek and Stark, 2016; Sadek et al., 2016). Studies suggested that H3R antagonists have shown promise in treating cognitive impairments associated with CNS disorders, such as AD, epilepsy, ADHD, narcolepsy (Bhowmik et al., 2012; Baronio et al., 2014), schizophrenia, Tourette syndrome, and ASD (Rapanelli and Pittenger, 2016; Eissa et al., 2018, 2020b, 2021, 2022b; Venkatachalam et al., 2021). Microglial activation and neuroinflammation are central to AD pathology. Targeting microglia via histamine regulation, especially through H3R inhibition, offers therapeutic potential. H3R antagonists can shift microglia from a proinflammatory (M1) to an anti-inflammatory (M2) state and reduce proinflammatory markers, supporting their role in AD treatment (Thomas et al., 2024a).

Scopolamine (SCO), a muscarinic cholinergic antagonist, is widely used in preclinical research to induce memory impairment and cognitive dysfunction in rodents. SCO induces amyloid-beta and tau overproduction, leading to mitochondrial oxidative stress and glutathione depletion in neurodegenerative diseases (Zhou et al., 2014) contributing to synaptic dysfunction, excitotoxicity, and memory impairment (Balaban et al., 2017). A study conducted by Cheon et al. (2021) showed that scopolamine-treated animals exhibit delirium-like behavioral patterns, along with biochemical and molecular signatures consistent with neuroinflammation and inflammasome activation. Acute administration of scopolamine (2 mg/kg, i.p.) significantly increased the levels of pro-inflammatory cytokines, particularly in the hippocampus and prefrontal cortex. Activation of the NLRP3 inflammasome and caspase-1 was also observed. RNA sequencing further confirmed that scopolamine altered gene expression profile related to neuro inflammation and nervous system development. Genes involved in cholinergic function (e.g., *Chrna3, Slc5a7, Slc38a5*) and inflammatory regulation (e.g., *Dhx58, Myo1f, Treml2*) were differentially expressed in scopolamine-treated mice. These results indicate that scopolamine-induced cognitive deficits are driven by disruptions in cholinergic signaling and activation of the inflammasome pathway.

Given the complex pathology of AD, targeting a single site is often insufficient for effective treatment. A promising approach is the development of MTDLs, which interact with multiple targets simultaneously. These compounds are designed with pharmacophore moieties that enable them to engage various receptors and enzymes, making them suitable for treating multifactorial diseases like AD (de Freitas Silva et al., 2018; Kumar et al., 2022). The integration of both ChE inhibition and H3R antagonism in one dual-active ligand may lead to substantial enhanced effects on improving cognitive capacity and memory. E100, dual-active ChEI and H3R antagonist has shown promising effects in ASD models by reducing repetitive behaviors, anxiety, and neuroinflammation (Eissa et al., 2019, 2020b). Hence, in the current study, the acute systemic administration of E100 at doses of 5, 10, and 15 mg/kg was evaluated using a series of behavioral and biochemical tests designed to assess various memory domains on a SCO-induced amnesia model in mice. The hippocampus and cerebellum were selected for biochemical investigations as both regions are strongly implicated in cholinergic regulation of memory and cognition which are central to the deficits modeled in this study. The hippocampus receives dense basal forebrain cholinergic projections and is essential for learning and memory, whereas the cerebellum contributes not only to motor coordination but also to higher cognitive and affective functions (Kruse et al., 2014; Chauhan et al., 2016; Maurer and Williams, 2017; Ananth et al., 2023; He et al., 2023).

Our results showed that E100 (10 and 15 mg/kg) significantly improved STM, whereas a dose of 5 mg/kg failed to significantly improve STM in NORT paradigm. However, all doses (5, 10, and 15 mg/kg) enhanced LTM, with the higher doses (10 and 15 mg/kg) of E100 being more effective, indicating a dose-dependent effect of E100 on memory function in NORT. Moreover, E100 (5, 10, and 15 mg/kg) significantly enhanced the percentage of alternation and counteracted SCO-induced deficits, as evidenced by increased spontaneous alternation in Y-maze test. E100’s dual mechanism, involving H3R heteroreceptor activity on glutamatergic and cholinergic neurons along with its ChE inhibitory effect, may enhance both working and recognition memory by modulating these pathways (Dere et al., 2003; Dash et al., 2007; Winters et al., 2008). This interaction likely underlies E100’s memory-enhancing effects in both NORT and YMT tasks. Moreover, the reference AChEI DOZ and the reference H3R antagonist PIT improved the STM and LTM as well as increased the percentage of alternation in both the NORT and Y-maze paradigms. DOZ, an AChEI improved recognition memory by increasing synaptic ACh availability, which plays a crucial role in object recognition and memory consolidation. Furthermore, PIT, a selective H3R antagonist, is known to increase histaminergic neurotransmission and enhance the release of other key neurotransmitters, such as dopamine, ACh, and glutamate. The effects of E100 on social memory were assessed using the TCT paradigm. While SCO-treated mice showed no change in innate sociability, they exhibited impaired social memory, as indicated by the observed reduced SNI value. However, systemic administration of E100 significantly improved social memory as evidenced by the observed increase in SNI in a dose-dependent manner relative to the SCO-treated group, and its effects were significantly higher than those observed for the reference AChEI, DOZ. These findings align with previous research, which reported that scopolamine impairs social memory without affecting innate sociability in mice, and that such deficits can be reversed by DOZ (Riedel et al., 2009). Moreover, this enhancement is likely due to E100’s dual mechanism, targeting both cholinergic and histaminergic systems. Our observed results for E100 are strongly supported by previous studies in which the critical role of cholinergic as well as histaminergic signaling in social memory was signified (Adolphs, 2001; Esbenshade et al., 2008).

In an additional behavioral paradigm, the effects of E100 on associative learning and memory applying the FCT task. Our observations for E100 showed a significant improvement of associative learning and memory in the cued and contextual fear conditioning tests in the SCO-induced dementia model. E100’s dual modulation of cholinergic and histaminergic pathways likely enhanced associative learning. Also, E100 may have enhanced memory through its dual action as ChEI and H3R antagonist, targeting multiple neurotransmitter systems involved in cognition. Our findings are in line with previous studies in which H3R antagonism increased glutamate, serotonin, and dopamine release, supporting synaptic plasticity and memory formation, while its ChEI activity was described to boost cholinergic signaling, therefore, counteracting dementia-related deficits (Passani and Blandina, 2011; Schliebs and Arendt, 2011). Hence, E100 exerts its cognitive-enhancing effects through a dual mechanism involving ChE inhibition and H3R antagonism. E100 inhibits ChE, leading to increased synaptic availability of acetylcholine, further supporting cholinergic signaling essential for attention, recognition memory, and social cognition. Concurrently, by antagonizing H3Rs, E100 modulates the release of multiple neurotransmitters—including acetylcholine, dopamine, glutamate, and histamine—thereby enhancing synaptic plasticity and neurotransmission critical for memory and learning. This synergistic modulation of cholinergic and histaminergic pathways underlies E100’s ability to improve short- and long-term memory, social recognition, and associative learning across diverse behavioral paradigms.

In the current experiments, E100 (10 and 15 mg/kg) reduced SCO-induced anxiety in the EPM test, confirming that its cognitive benefits are due to memory enhancement and are not confounded by emotional or anxiolytic effects. The effect of E100 on locomotor activity has been measured in a previous study using the open-field test in VPA-induced ASD in C57BL/6 mice. The study analyzed total distance traveled, an index of locomotor activity as well as the time spent in the center and periphery. The results showed that E100 did not significantly affect the total distance traveled and time spent in the periphery, indicating that the anxiolytic-like effects observed (i.e., increased time spent in the center) were not confounded by changes in locomotion (Eissa et al., 2019). To further comprehend the previous findings for E100, an abrogation study was conducted to clarify the role of histaminergic neurotransmission in observed improvements in memory brought by E100 by co-administering it with the CNS-penetrant and selective H3R agonist RAMH. The resulting decline in memory performance across all the behavioral paradigms confirms that E100’s effects were mediated, at least in part, through H3R antagonism. Notably, the 10 mg/kg dose was selected as the optimal dose based on its comparable efficacy to the 15 mg/kg dose across the majority of the conducted tests. Given that both doses demonstrated similar outcomes, the lower dose (10 mg/kg) was chosen to minimize potential side effects while maintaining therapeutic effectiveness. Consequently, all subsequent biochemical studies and the abrogation tests were performed using the 10 mg/kg dose.

Neuroinflammation is a key pathological hallmark of cognitive impairment (Carthy and Ellender, 2021). Proinflammatory cytokines released from microglia has been linked to progression of AD (Wang et al., 2015). A study revealed that Aβ aggregates activate the NLRP inflammasome in neurons, leading to caspase-1 activation and pyroptosis—an inflammatory form of programmed cell death. This process releases IL-1β and IL-18, worsening neuroinflammation and contributing to neuronal loss, highlighting caspase-1’s key role in AD progression (Wójcik et al., 2024). This study evaluated caspase-1 activity in the hippocampus and cerebellum to assess scopolamine (SCO)-induced neuroinflammation and the neuroprotective effects of pharmacological interventions. The hippocampus and cerebellum were selected for further studies as both are critically involved in cognitive functions such as memory and learning. SCO significantly increased caspase-1 activity in both areas, indicating heightened inflammatory and apoptotic signaling. However, treatment with E100 (10 mg/kg) markedly reduced caspase-1 levels, with its efficacy surpassing that of both reference drugs DOZ and PIT. This suggests that E100 exerts potent anti-inflammatory and neuroprotective effects, likely through modulation of the inflammasome pathway, thereby preserving memory and cognitive function. To further examine its anti-inflammatory potential, E100’s impact on TNF-α and IL-1β levels was measured. E100 significantly decreased both cytokines in the hippocampus and cerebellum of SCO-treated mice. These results support E100’s role in reducing neuroinflammation, which is strongly linked to cognitive decline in neurodegenerative diseases like AD, highlighting its potential to slow disease progression.

**FIGURE 12:**
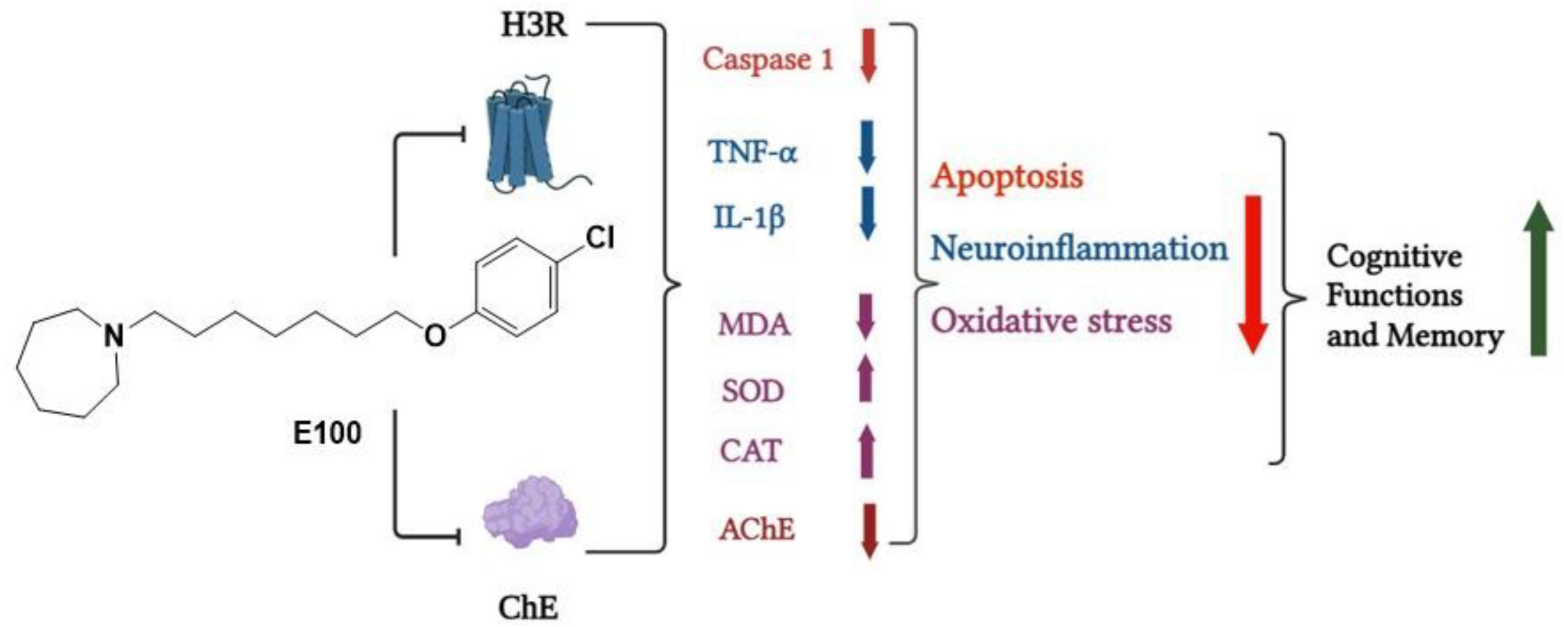
Effects of the dual-active ChEI and H3R antagonist E100 on apoptosis, neuroinflammation, AChE activity, and oxidative stress in the hippocampus and cerebellum of scopolamine-induced amnesic mice and consequent improvement in cognitive functions and memory.

Dementia has also been linked to the excessive production of ROS, resulting from the disruption of cellular antioxidant systems (Kamat et al., 2016). Lipid peroxidation is a critical pathological process in brain tissues, arising from an imbalance between the production of ROS and the efficiency of the body’s natural antioxidant defense mechanisms (Eissa et al., 2018). Biochemical analysis revealed that SCO treatment increased brain MDA levels and reduced GSH and SOD, indicating oxidative stress. Treatment with E100 reversed these effects by reducing MDA and restoring GSH and SOD levels, suggesting enhanced antioxidant defense. Interestingly, the co-administration of the H3R agonist, RAMH with the optimal dose of E100, diminished the observed E100’s protective effects against elevated pro-inflammatory cytokines, oxidative stress markers and caspase 1 levels further indicating that histaminergic neurotransmission is crucial for E100’s neuroprotective action in SCO-induced amnesia. The cholinergic enhancement induced by E100 in SCO-treated mice was supported by the evaluation of AChE activity in hippocampus and cerebellum of SCO-treated mice. Systemic administration of E100 (10 mg/kg) significantly reduced AChE activity in SCO-treated mice, showing a comparable effect to that of the reference drug DOZ, a well-established AChE inhibitor leading to improved cognitive capacity and memory. This study investigated the effects of E100, a dual ChEI and H3R antagonist, on SCO induced memory impairment in male C57BL/6 mice. Behavioral tests (NORT, EPM, Y-Maze, TCT, FCT) showed that E100 (5–15 mg/kg, i.p.) significantly improved short-term, long-term, and spatial memory, as well as reduced anxiety-like behavior. E100 (10 mg/kg, i.p.) significantly reduced neuroinflammation and oxidative stress markers while caspase-1 activity was decreased (Figure 12). In several behavioral paradigms, E100 showed numerically greater improvements than H3R inverse agonist, PIT; however, these differences did not reach statistical significance. While these may indicate a potential advantage for E100, additional studies with larger sample sizes are required. Compared with PIT, E100 produced more pronounced reductions in oxidative stress markers and greater suppression of neuroinflammatory mediators such as TNF-α, IL-1β, and caspase-1. Overall, E100 demonstrated superior efficacy to Pitolisant in the behavioral and biochemical assessments, suggesting enhanced therapeutic potential (Table 1).

**TABLE 1:** Comparative summary of E100 and Pitolisant.

Test/parameter	E100	PIT	Summary
DI (STM)	↑	↑	PIT > E100
DI (LTM)	↑	↑	E100 > PIT
Time spent in open arms (anxiety)	↑	↑	PIT > E100
Alternation	↑	↑	E100 > PIT
Sociability Index	↑	↑	E100 > PIT
Social Novelty Index	↑	↑	E100 > PIT
Freezing (contextual/cued)	↑	↑	E100 > PIT
Caspase-1 activity	↓	↓	E100 > PIT
Oxidative stress			
SOD	↑	↑	E100 > PIT
GSH	↑	↑	
MDA	↓	↓	
Neuroinflammatory markers (TNF-α, IL-1β)	↓	↓	E100 > PIT

↑ Denotes increase, ↓ denotes decrease.

Although no direct blood–brain barrier (BBB) permeability data are currently available for E100, its centrally mediated behavioral effects indicate that it is able to access the brain. In particular, the abrogation of E100-induced behavioral enhancement by the CNS-penetrant and selective H3R agonist (R)-α-methylhistamine (RAMH, 10 mg/kg, i.p.) indicates that E100 exerts its effects through central H3R antagonism. This indirect evidence suggests that E100 is capable of crossing the BBB and acting within the central nervous system. Nevertheless, further BBB permeability studies are required, to confirm and quantify its brain penetration.

## 5 Conclusion

The findings of the present study underscore the significant potential of dual-acting ChEIs and H3R antagonists as a promising therapeutic strategy for modulating memory impairment and enhancing cognitive function. The results demonstrate that acute systemic administration of E100 markedly improves cognitive performance, reducing apoptosis, neuroinflammation and oxidative stress as summarized in Figure 12. Furthermore, the study provides compelling evidence that simultaneously targeting two distinct neuronal pathways, namely cholinergic systems and H3Rs, yields pharmacological and behavioral outcomes that are superior to those achieved by targeting either pathway in isolation. This supports the therapeutic potential of dual-acting H3R antagonists and AChEIs for neuropsychiatric disorders, particularly those characterized by impairments across multiple domains of memory processing, such as AD and other forms of dementia.

## 6 Limitations

This study primarily investigated the acute effects of E100, without assessing long-term outcomes or potential side effects, an important limitation when evaluating treatments for chronic conditions like AD. Although we demonstrated changes in IL-1β and caspase-1, we did not assess upstream regulators such as NLRP3, representing an additional limitation. Further studies are warranted to investigate both upstream NLRP3 inflammasome activation and downstream signaling pathways to provide a more complete mechanistic understanding of E100’s anti-inflammatory and neuroprotective effects. The use of a scopolamine-induced amnesia model reproduces cholinergic dysfunction but does not fully capture hallmark AD pathologies such as Aβ plaques and tau tangles. Future studies in disease-relevant animal models, such as transgenic models of neurodegeneration, are needed to assess its therapeutic potential and safety profile.

## Data Availability

The original contributions presented in the study are included in the article, further inquiries can be directed to the corresponding author.
